# Genomic and immune profiling of prognostic risk groups in IgM gammopathy reveals novel biomarkers beyond *MYD88* L265P

**DOI:** 10.3389/fimmu.2025.1604089

**Published:** 2025-07-01

**Authors:** David F. Moreno, Ferran Nadeu, Fara Brasó-Maristany, Sergi Vaqué, Sara Paz, Joan Mañé, Oriol Cardús, Elena Medina, Ester Lozano, Luis Gerardo Rodríguez-Lobato, Anna de Daniel, Natalia Tovar, María T. Cibeira, Joan Bladé, Laura Rosiñol, Aleix Prat, Dolors Colomer, Carlos Fernández de Larrea

**Affiliations:** ^1^ Amyloidosis and Myeloma Unit, Department of Hematology, Hospital Clínic de Barcelona, University of Barcelona, Barcelona, Spain; ^2^ Institut d’Investigacions Biomèdiques August Pi i Sunyer (IDIBAPS), University of Barcelona, Barcelona, Spain; ^3^ Centro de Investigación Biomédica en Red de Cáncer (CIBERONC), Madrid, Spain; ^4^ Institute of Cancer and Blood Diseases, Barcelona, Spain; ^5^ Reveal Genomics, Barcelona, Spain; ^6^ Hematopathology Unit, Department of Pathology, Hospital Clínic de Barcelona, University of Barcelona, Barcelona, Spain; ^7^ Breast Cancer Unit, Institute of Oncology (IOB)-QuirónSalud, Barcelona, Spain

**Keywords:** MGUS, Waldenström, *MYD88*, CXCR4, immune

## Abstract

**Background:**

*MYD88* L265P is an early mutation in IgM monoclonal gammopathy of undetermined significance (MGUS) and asymptomatic Waldenström macroglobulinemia (WM). Given the high prevalence of the *MYD88* mutation observed in epidemiological studies, its presence is not sufficient to drive disease progression. In fact, a recent risk model of progression reported that the impact of other laboratory biomarkers was superior to the *MYD88* mutation’s presence. Due to the low incidence of these clinicopathological entities, there is a need for a better characterization of tumor and immune cells that can help to identify novel biomarkers. We hypothesize that the characterization of the risk groups in asymptomatic patients could improve the discovery of drivers of disease progression

**Methods:**

We characterized the genomic and immune landscape of the most recent prognostic risk categories in 19 IgM MGUS and 17 asymptomatic WM patients. We performed targeted next generation sequencing (NGS) on CD19+ cells from bone marrow samples at diagnosis using a panel of 54 lymphoma-driver genes. Whole bone marrow samples were also used to measure mRNA gene expression in tumor and immune cells using the PanCancer ImmuneProfiling panel on the nCounter platform (NanoString).

**Results:**

We observed that low-risk patients were only characterized by the presence of *MYD88* L265P, while intermediate- and high-risk groups harbored additional mutations on *CXCR4*, *KMT2D*, *ARID1A* and *EP300*. Regarding the mRNA expression analyses, we found an increased proportion of myeloid cells in the low-risk group, with monocytes having a significant decrease in low versus high-risk patients. The high-risk group also upregulated genes involved in the activation of NF-κB and B-cell receptor (BCR) signaling, while low-risk patients upregulated genes associated with an alternative activation of B cells or a decrease of the BCR signaling, such as TOLLIP, CEACAM1 and CR1.

**Conclusions:**

Beyond the *MYD88* mutation, we described novel molecular mechanisms associated with high-risk patients, as an effort moving towards easy-to use new biomarkers in IgM gammopathy.

## Introduction

IgM monoclonal gammopathy of undetermined significance (MGUS) and Waldenström macroglobulinemia (WM) are characterized by the presence of a monoclonal IgM in serum produced by abnormal mature B cells located in the bone marrow (BM) (1, 2). The malignant B-cell clone is proportionally higher in WM compared to IgM MGUS; however, they share similar immunophenotypical and genomic characteristics. For instance, it has been described that both share the expression of CD22low and CD25+ on abnormal B cells (3, 4), along with mutations in *MYD88* and *CXCR4 (* 5–8). In this sense, intensive research to categorize the risk of progression have made possible to model the progression free survival (PFS) in both IgM MGUS and WM using common biomarkers from routine clinical practice (9–12). Regarding the *MYD88* L265P impact, previous reports suggested that wild type (wt) patients, either asymptomatic or symptomatic, had worse outcomes (10, 13, 14). More recently, its independent value was put into debate in two multicenter studies, in which the mutation was not an independent predictor in asymptomatic patients (11), or did not impact in the overall survival of symptomatic patients (12). In line with these findings, the mutation burden analyzed with droplet digital polymerase chain reaction (ddPCR) showed that a high MYD88 mutation burden was associated with higher risk of progression, rather than the presence or absence of the mutation (7).

Therefore, prognostic risk models in IgM gammopathy should include a comprehensive analysis to further improve biomarker discovery. Single cell technologies have improved the field in characterizing with great detail the cell-of-origin of the lymphoplasmacytic clones and the tumor microenvironment (TME) in IgM MGUS and WM (15–19); however, its application in the clinical setting remains challenging, as well as its prognostic validation. In the present study, we hypothesized that the addition of other somatic mutations beyond *MYD88* L265P and gene expression patterns of the clonal B cells and TME using affordable, high throughput techniques could characterize the clinical phenotypes behind the prognostic risk categories in asymptomatic patients and could serve as biomarkers of disease progression.

We characterized the clinical risk categories based on the most recent prognostic classification that relied on the presence of a serum IgM ≥ 10 g/L, BM infiltration ≥ 20%, β2-microglobulin ≥ 3 mg/L and albumin < 4 g/dL. High-risk patients had at least 3 factors, while intermediate- and low-risk groups had 2, and none or 1 risk factors, respectively (11). We used a panel of genes that are recurrently altered in mature B-cell lymphomas to sequence the tumor cells of IgM gammopathy patients, followed by the characterization of the TME in response to the malignant clone, resembling the actual BM environment. We found that *CXCR4*, *ARID1A*, *KMT2D* and *EP300* mutations are present in intermediate and high-risk groups, also showing an association with the B-cell receptor (BCR) signaling. In contrast, low risk patients harbored only *MYD88* L265P and showed upregulation of genes that may interfere with BCR activation. These biomarkers can explain the differences of progression risk in IgM gammopathy patients and could be further exploited in the clinic.

## Methods

### Patients and sample processing

We analyzed patients with IgM MGUS and asymptomatic WM who were diagnosed between 2021 and 2022 in Hospital Clínic de Barcelona. The diagnosis was established according to the International Consensus criteria (2). All patients had bone marrow aspirates available, and only WM patients underwent a bone marrow biopsy. WM patients were not exposed to previous treatments. All patients signed an informed consent to collect BM samples in the biobank of Hospital Clínic de Barcelona, in accordance with the Declaration of Helsinki. The study was approved by the institutional review board.

The methodology followed to process the samples is described in the **Supplementary Material**. In short, BM samples were collected in EDTA tubes for further characterization of B cells and plasma cells using multiparametric flow cytometry (MFC) (**Supplementary Table 1**), detection of the *MYD88* L265P by ddPCR, targeted next generation sequencing (NGS) and mRNA expression analyses. Regarding the MFC gating strategy, CD4+, CD8+ and NK cells were quantified relative to the total CD3+ T-cell population or events. B cells and plasma cells were quantified relative to the total number of events (bone marrow cellularity). NGS was performed after CD19+ isolation by immunomagnetic beads (Milteny Biotec, Bergisch Gladbach, Germany). RNA was extracted from whole BM samples after three steps of erythrocyte lysis, centrifugation followed with the supernatant discarded to capture the signal from all immune cells.

### Next generation sequencing of target genes

DNA from the CD19+ enriched samples was used for NGS using a panel of 54 target genes (**Supplementary Table 2**) that are recurrently mutated in mature B cell neoplasms (SOPHiA Lymphoma Solution, SOPHiA Genetics, Rolle, Switzerland). A minimum of 100 ng of DNA was used for library preparation, following the recommendations of SOPHiA Genetics. Libraries were then sequenced using 150 bp paired end reads on a NextSeq 2000 P1 instrument (Illumina, San Diego, CA, USA), with a mean coverage of 1500x. The analysis of the sequencing data is described in the **Supplementary Material**.

### Quantification of gene expression

The isolated RNA was quantified using Qubit 2.0 Fluorometer (Thermo Fisher). At least 300 ng of RNA per sample was used in the nCounter instrument (NanoString Technologies, Inc. Seattle, WA). The panel used was the PanCancer ImmuneProfiling which consisted of 730 genes that are involved in adaptive and innate immune response to cancer and 40 housekeeping genes. The analysis of the mRNA expression is described in the **Supplementary Material**.

### Statistical analyses

The chi-square test was used to compare proportions across clinical categories. The Kruskal-Wallis test was used to compare quantitative features according to diagnosis. Pearson correlation or Spearman tests were used to obtain a correlation index between numerical values. The log-rank test was used to assess the significance between conditions in PFS, and the Kaplan-Meier method to plot survival curves. For differential gene expression (DGE) analysis, a false discovery rate (q-value) of less than 0.25 was considered significant. All downstream analyses were performed using R (v. 4.4.0, R Foundation for Statistical Computing, Vienna, Austria).

## Results

### Clinical characteristics

**Figure 1:**
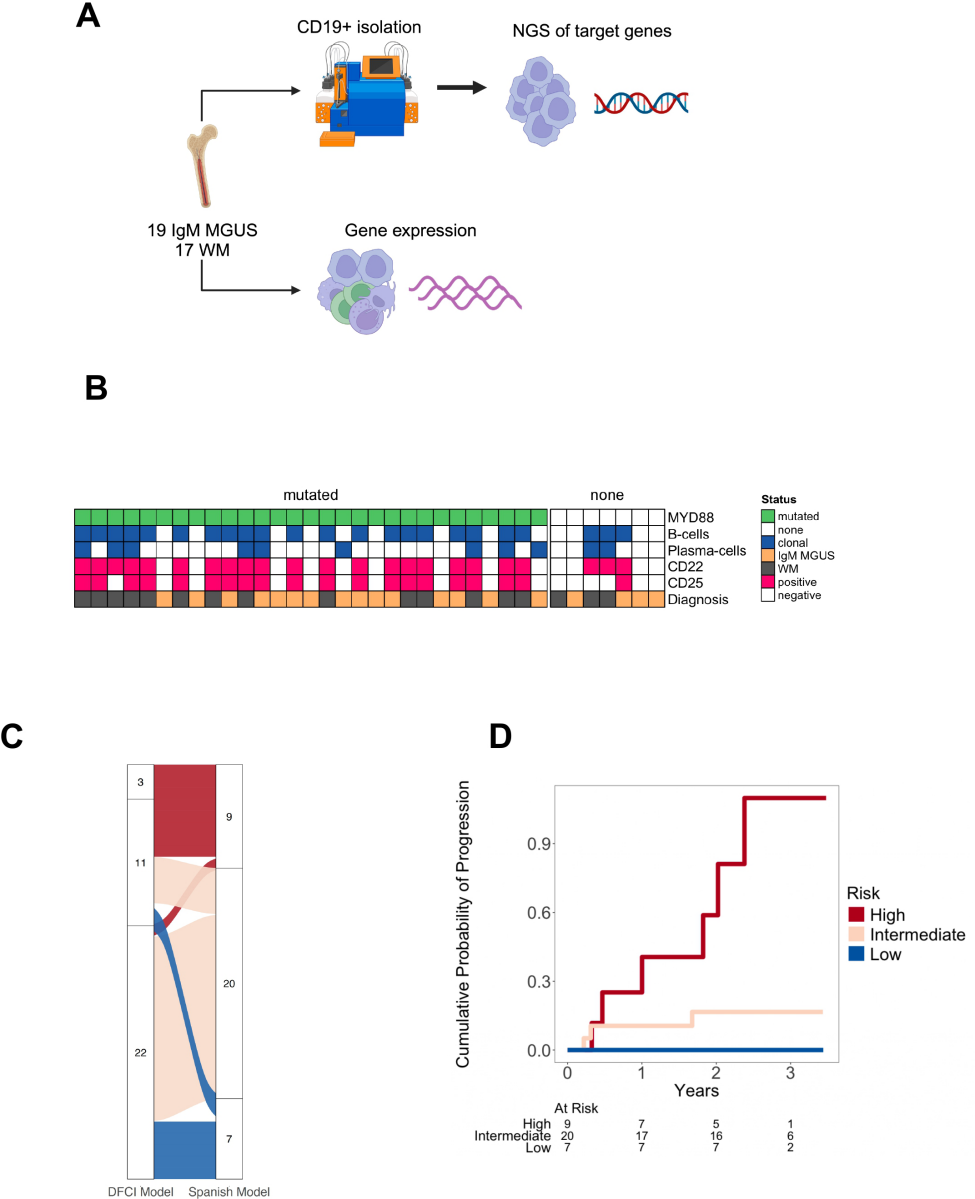
Sample processing and baseline patient’s characteristics. **(A)** Whole bone marrow samples were processed with immunomagnetic sorting beads to obtain CD19+ cells followed by next generation sequencing (NGS) targeting 54 lymphoma driver genes. mRNA was obtained from whole bone marrow samples for further use in the nanoString nCounter platform using the PanCancer immuneprofiling assay. **(B)** Immunophenotypical characteristics of IgM monoclonal gammopathy of undetermined significance (MGUS) and Waldenström macroglobulinemia (WM) according to the presence of *MYD88* L265P detected by droplet digital polymerase chain reaction. **(C)** Distribution of patients based on the risk categorized by the Dana Farber Cancer Institute (DFCI) and the Spanish models. **(D)** Probability of progression based on risk categories according to the Spanish model.

A total of 36 patients (19 IgM MGUS and 17 WM) were evaluated. **Table 1** summarizes their clinical characteristics. WM patients exhibited higher levels of both serum IgM and bone marrow lymphocytic infiltration, along with lower serum albumin levels compared to those with IgM MGUS. In addition, WM patients had a trend towards lower serum IgG and higher β2-microglobulin levels than IgM MGUS. The majority of IgM MGUS and WM patients had kappa-light chain restriction, and no differences were observed regarding the plasma cell bone marrow infiltration between the two groups. All patients with WM had a clonal B-cell population detected in the BM except for one who was diagnosed with lymphoplasmacytic lymphoma in the lymph nodes. On the contrary, only 7 (36.8%) patients with IgM MGUS had a B-cell clone in the BM. The size of the B-cell clones measured by MFC was higher in WM compared to IgM MGUS (24.5% vs. 85.5%; p<0.001). We also assessed the expression of antigen markers on B-cell clones that are commonly associated with IgM gammopathy. In fact, the presence of CD22^low^ (36.8% vs. 94.1%, p<0.001) and CD25+ (36.8% vs. 76.5%, p=0.017) were detected in both IgM MGUS and WM patients. Two patients with IgM MGUS had only clonal plasma cells without any detectable B cell clone by MFC. Regarding the *MYD88* L265P detection using ddPCR or targeted NGS, the mutation was detected in 15 (78.9%) and 14 (82.4%) patients with IgM MGUS and WM, respectively. The mean MYD88 mutation burden assessed by ddPCR was 1.22% and 8.63% in patients with IgM MGUS and WM (p<0.001), respectively. The *MYD88* mutation burden was partially correlated with the proportion of clonal B cells (r=0.54, p<0.001) and plasma cells (r=0.40, p=0.024) in the BM detected by MFC. Seven (36.8%) *MYD88* mutated IgM MGUS patients did not have any detectable clonal B or plasma cells by MFC. **Figures 1A, B** summarizes the processing of samples and associations between the MYD88 mutation status and MFC. According to the Spanish risk model, 78.9% and 82.4% of IgM MGUS and WM patients were categorized as intermediate or high risk, respectively. The *MYD88* mutation was prevalent in 12 (80.0%) out of 15 intermediate and high-risk IgM MGUS, and 11 (78.6%) out of 14 intermediate and high-risk WM. In total, six high-risk and 3 intermediate-risk patients progressed. On the other hand, the DFCI risk model classified most patients as low and intermediate risk, with 3 high-risk, 4 intermediate-risk and 2 low-risk patients experiencing disease progression (**Figure 1C**). These findings led us to use the Spanish model as the framework for further analyses. After a median follow-up of 2.62 years (interquartile range [IQR] 2.32 – 3.10), high-risk patients had an increased cumulative probability of progression compared to intermediate- (p=0.013) and low-risk patients (p=0.010) (**Figure 1D**). Seven WM and 2 IgM MGUS patients experienced disease progression.

**Table 1:** Patients’ characteristics.

Patients characteristics	IgM MGUS *N*=19	WM *N*=17	*P*-value
Age, median (IQR)	67.6 (54.1 – 75.7)	71.7 (68.4 – 74.0)	0.4
Sex, female (%)	11 (58)	11 (65)	0.7
Serum M-protein, g/L	2.8 (0.0 – 4.1)	6.9 (4.3 – 9.9)	<0.001
Albumin, g/dL	4.5 (4.3 – 4.6)	4.2 (3.9 – 4.4)	0.012
β2-microglobulin mg/L	1.9 (1.4 – 3.2)	2.5 (2.2 – 3.2)	0.07
Serum IgM, g/L	4.0 (2.8 – 5.0)	6.7 (3.3 – 14.2)	0.03
Serum IgG, g/L	8.4 (7.4 – 10.1)	5.9 (4.8 – 9.8)	0.08
Serum IgA, g/L	1.5 (0.9 – 1.7)	1.1 (0.6 – 1.6)	0.2
Bone marrow, (%) Lymphocytes Plasma cells	11 (7 – 14) 2 (1 – 4)	26 (14 – 32) 2 (1 – 4)	0.001 0.9
Kappa light chain isotype, (%)	14 (74)	13 (76)	0.9
*MYD88* L265P by ddPCR, (%)	15 (78.9)	14 (82.4)	0.5
Risk category, (%) Low Intermediate High	4 (21.1) 13 (68.4) 2 (10.5)	3 (17.6) 7 (41.2) 7 (41.2)	0.10

The whole series was composed of 19 patients diagnosed with IgM monoclonal gammopathy of undetermined significance (MGUS) and 17 with Waldenström macroglobulinemia (WM). There were no missing values. N, number of patients; IQR, interquartile range; M-protein, serum monoclonal protein.

### Genomic landscape of prognostic risk categories

Regarding the *MYD88* status, there was no association between risk groups and the mutation prevalence (85.7%, 80.0%, 77% for low-, intermediate-, and high-risk, respectively; p=0.920) assessed either by ddPCR or NGS. The mean MYD88 mutation burden by ddPCR was 6.0%, 1.80% and 9.9% for low-, intermediate-, and high-risk, respectively (p=0.065). The Pearson correlation between the *MYD88* L265P VAF detected by NGS and the mutation burden by ddPCR was 0.82 (p<0.001) (**Supplementary Figure 1A**). A total of 93 mutations were detected in 34 (94.4%) patients using NGS (**Supplementary Table 3**). Two patients without detectable mutations by NGS were one IgM MGUS and one WM, and both were categorized as intermediate risk. The first one had a *MYD88* L265P mutation burden by ddPCR of 0.12%, while the second one did not have the mutation by ddPCR. Based on diagnosis, 14 (73.7%) IgM MGUS and 14 (82.4%) WM patients had the *MYD88* L265P mutation by NGS (n=36). The mean VAF of the *MYD88* L265P by NGS was 7.81% and 29.3% in IgM MGUS and WM, respectively.

**Figure 2:**
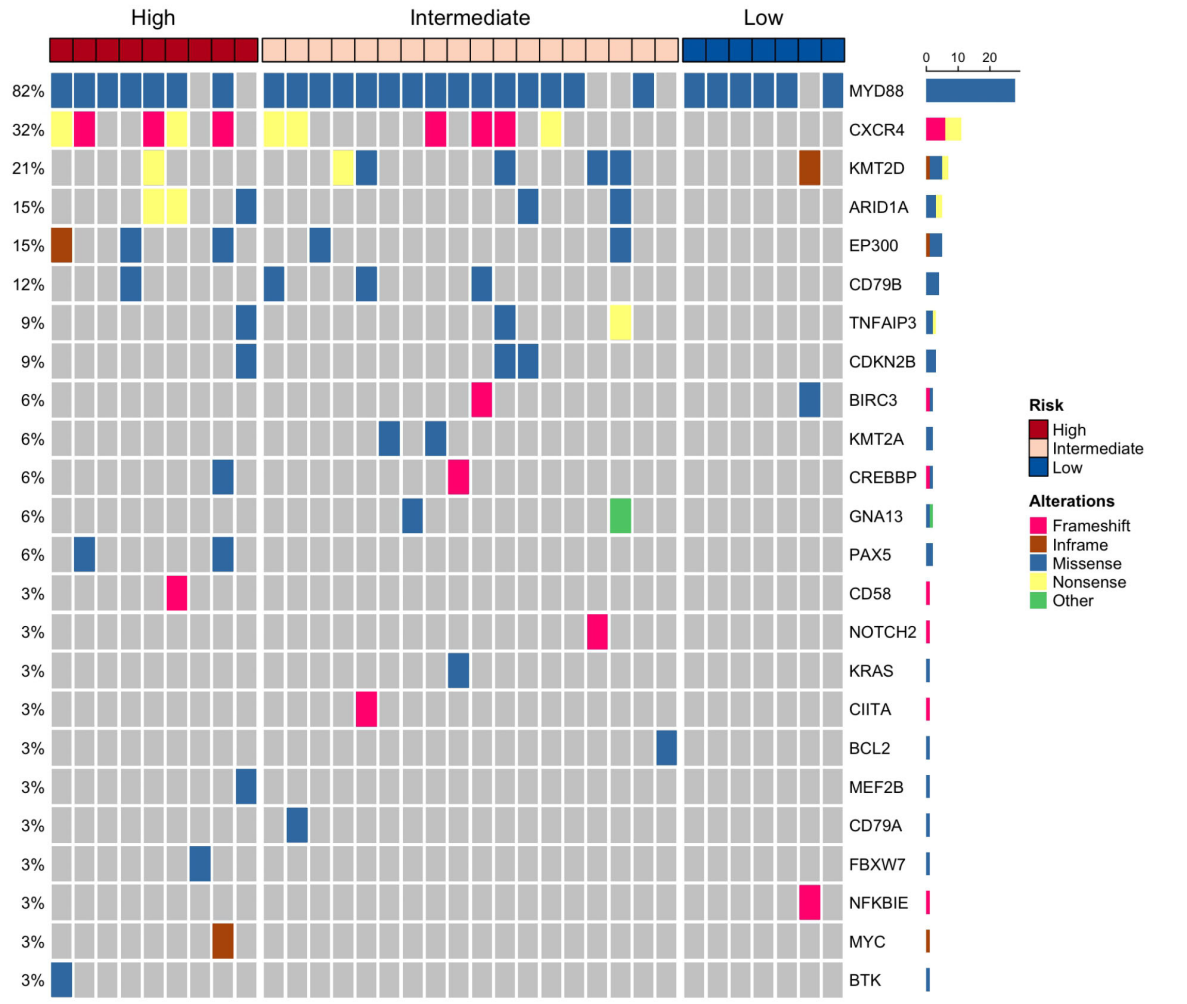
Single nucleotide variants and small indels detected by targeted next generation sequencing (NGS) in patients with IgM monoclonal gammopathy of undetermined significance (MGUS) and Waldenström macroglobulinemia (WM). In total, 34 out of 36 patients harbored mutations in driver genes by NGS.

The second most frequent mutation involved was *CXCR4*, in which 4 (21.1%) IgM MGUS and 7 (41.1%) WM patients had at least one mutation. The mean VAF of *CXCR4* mutations was 1.7% and 12.0% in IgM MGUS and WM, respectively. *KMT2D* mutations were prevalent in 4 (11.1%) IgM MGUS and 3 (8.3%) WM patients. Other less frequent mutations had an uneven distribution. For instance, mutations in *BIRC3*, *BCL2*, *CIITA*, *KRAS*, *MEF2B*, *NFKBIE* and *NOTCH2* were only detected in IgM MGUS (**Supplementary Figure 1B**). The mutations in *CXCR4* were detected only in intermediate- and high-risk groups, harboring also the *MYD88* mutation. *CXCR4* mutations encompassed frameshift and nonsense alterations such as c.1025C>A; p.Ser342* or c.1012C>T; p.Arg338*, the latter exclusively found in WM. One WM patient who was categorized as intermediate-risk presented a mutation in *KMT2D* and had disease progression, while 3 had IgM MGUS and were intermediate-risk without presenting progression. *ARID1A* was altered in 5 (13.8%) patients, being all categorized as intermediate and high risk. Three patients who had *ARID1A* mutations presented disease progression. *EP300* was the fifth gene most frequently altered, and the mutations were only present in intermediate and high-risk groups. EP300 c.2091T>G, previously reported in diffuse large B cell lymphoma (DLBCL) (20), was found in one IgM MGUS and one WM. No recurrent somatic mutations were found in *KMT2D*, *ARID1A* or *EP300* in patients who progressed (**Table 2**). Regarding the number of mutations, high-risk patients had a median of 4 somatic mutations, whereas intermediate- and low-risk patients had only 3 and 2 median somatic mutations (p=0.014) (**Figure 2**).

**Table 2:** Somatic mutations (single nucleotide variants and small indels) in patients who progressed.

Patient	Diagnosis	Risk	Chromosome	Position	Reference allele	Variant allele	Gene	Coding sequence	Protein sequence	VAF (%)
PNT1	WM	High	2	136114915	G	T	*CXCR4*	c.1025C>A	p.Ser342*	10.1
PNT1	WM	High	22	41178506	TCAG	T	*EP300*	c.6798_6800delGCA	p.Gln2267del	41.2
PNT1	WM	High	3	38141150	T	C	*MYD88*	c.779T>C	p.Leu260Pro	11.1
PNT1	WM	High	X	101356177	A	G	*BTK*	c.1543T>C	p.Cys515Arg	10.3
PNT1	WM	High	X	101356177	A	T	*BTK*	c.1543T>A	p.Cys515Ser	0.9
PNT5	WM	High	3	38141150	T	C	*MYD88*	c.779T>C	p.Leu260Pro	42.5
PNT29	WM	High	4	152328358	C	A	*FBXW7*	c.1268G>T	p.Gly423Val	0.5
PNT26	WM	High	1	26774683	C	T	*ARID1A*	c.4456C>T	p.Gln1486*	65.9
PNT26	WM	High	1	116544389	TTAAGTTGTAGA	T	*CD58*	c.275_285delTCTACAACTTA	p.Ile92fs	0.4
PNT26	WM	High	2	136114928	G	A	*CXCR4*	c.1012C>T	p.Arg338*	36.8
PNT26	WM	High	3	38141150	T	C	*MYD88*	c.779T>C	p.Leu260Pro	66.0
PNT30	WM	Intermediate	1	26731539	C	A	*ARID1A*	c.1738C>A	p.Pro580Thr	0.8
PNT30	WM	Intermediate	1	26732787	T	G	*ARID1A*	c.1915T>G	p.Leu639Val	18.5
PNT30	WM	Intermediate	12	49048002	C	T	*KMT2D*	c.4199G>A	p.Cys1400Tyr	1.1
PNT30	WM	Intermediate	17	65014258	C	T	*GNA13*	c.1133G>A	p.Ter378Ter	50.3
PNT30	WM	Intermediate	22	41146776	T	G	*EP300*	c.2091T>G	p.Ser697Arg	44.1
PNT30	WM	Intermediate	22	41170438	C	T	*EP300*	c.4319C>T	p.Pro1440Leu	18.6
PNT30	WM	Intermediate	6	137874967	TTCCGCTGGC	T	*TNFAIP3*	c.419_427delTCCGCTGGC	p.Phe140_Gln143delinsTer	21.4
PNT32	WM	High	16	3731781	T	C	*CREBBP*	c.4885A>G	p.Lys1629Glu	0.9
PNT32	WM	High	2	136114911	AGATGAATGTCCACCTCG	A	*CXCR4*	c.1012_1028delCGAGGTGGACATTCATC	p.Arg338fs	0.5
PNT32	WM	High	22	41150154	C	A	*EP300*	c.2773C>A	p.Pro925Thr	16.0
PNT32	WM	High	3	38141150	T	C	*MYD88*	c.779T>C	p.Leu260Pro	1.1
PNT32	WM	High	8	127738358	C	CCAG	*MYC*	c.154_156dupCAG	p.Gln52dup	6.4
PNT32	WM	High	9	37006497	C	T	*PAX5*	c.451G>A	p.Val151Ile	2.7
PNT35	IgM MGUS	High	1	26766292	A	G	*ARID1A*	c.2804A>G	p.Asn935Ser	1.2
PNT35	IgM MGUS	High	19	19146330	A	G	*MEF2B*	c.845T>C	p.Leu282Pro	0.2
PNT35	IgM MGUS	High	6	137874871	A	G	*TNFAIP3*	c.322A>G	p.Thr108Ala	1.9
PNT35	IgM MGUS	High	9	22008816	G	T	*CDKN2B*	c.138C>A	p.Phe46Leu	5.9
PNT27	IgM MGUS	Intermediate	3	38141150	T	C	*MYD88*	c.779T>C	p.Leu260Pro	0.7

MGUS, monoclonal gammopathy of undetermined significance; WM, Waldenström macroglobulinemia; VAF, variant allele frequency.

### Immune cells infiltration

**Figure 3:**
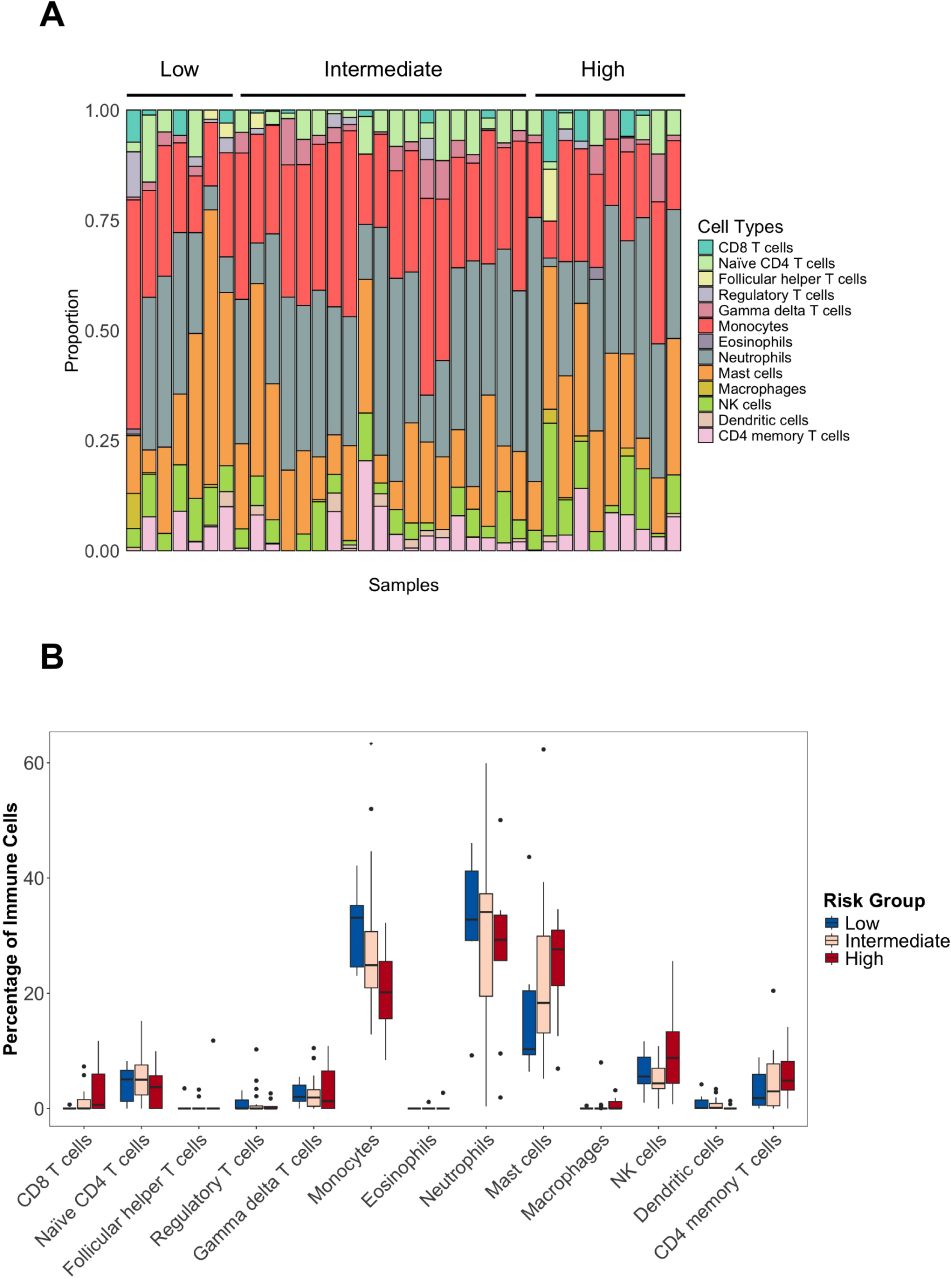
Immune cell types in IgM gammopathy. **(A)** Proportion of the cells across risk categories. B-cells and plasma cells were removed to illustrate the tumor microenvironment cells. **(B)** Distribution of cell types, showing the decrease of monocytes from low- to high-risk groups (* p<0.05).

We next examined the immune cell landscape. We used a previously validated gene signature to deconvolute cell types from our targeted gene expression panel, similar to other studies in solid tumors and DLBCL (21, 22). The proportion of CD8+ (r=0.36; p=0.033), NK (r=0.42; p=0.010) and CD4+ T cells (r=0.37; p=0.030) inferred from gene expression were positively correlated with that obtained from MFC (**Supplementary Figures 2A-C**). We excluded the inferred proportion of B cells and plasma cells to better illustrate the role of immune cells, and merged specific cell types (mast cells, macrophages, NK cells, dendritic cells and CD4 T cells) that contained multiple subtypes to increase the correlation of canonical genes from our targeted mRNA assay and the reference. We found high heterogeneity of the immune cell infiltration in each risk group, characterized by a high proportion of myeloid cells. Meanwhile, the T-cell subpopulations represented a few percentage of the immune cell landscape in each patient. For instance, the mean infiltration rates for CD8+ T cells, monocytes, mast cells and neutrophils were 1.4%, 26.1%, 21.3% and 29.7%, respectively. Among all cell types, the monocyte infiltration showed a consecutive significant decrease from low towards high-risk patients (p=0.040). Of note, mast cells had an increasing trend towards high risk and represented the third most abundant immune cell type (mean of low-, intermediate-, and high-risk groups were 17.1%, 21.5% and 24.4%, respectively) (**Figures 3A, B**). According to diagnosis, the proportion of monocytes, neutrophils and mast cells were similar in both IgM MGUS and WM. Although present in low counts, naïve CD4+ T cells (p=0.022) and dendritic cells (p=0.037) were increased in IgM MGUS patients. We further investigated whether the presence of the *MYD88* L265P or *CXCR4* mutations were associated with a distinct immune repertoire. The frequency of CD8+ T cells (p=0.017), NK cells (p=0.048) and mast cells (p=0.015) were increased in the *MYD88* mutated samples, whereas the neutrophils (p=0.049) proportion was decreased (**Supplementary Figures 2D, E**). *CXCR4* mutations was not associated with any particular cell type (data not shown).

### Differentially expressed genes across risk categories

**Figure 4:**
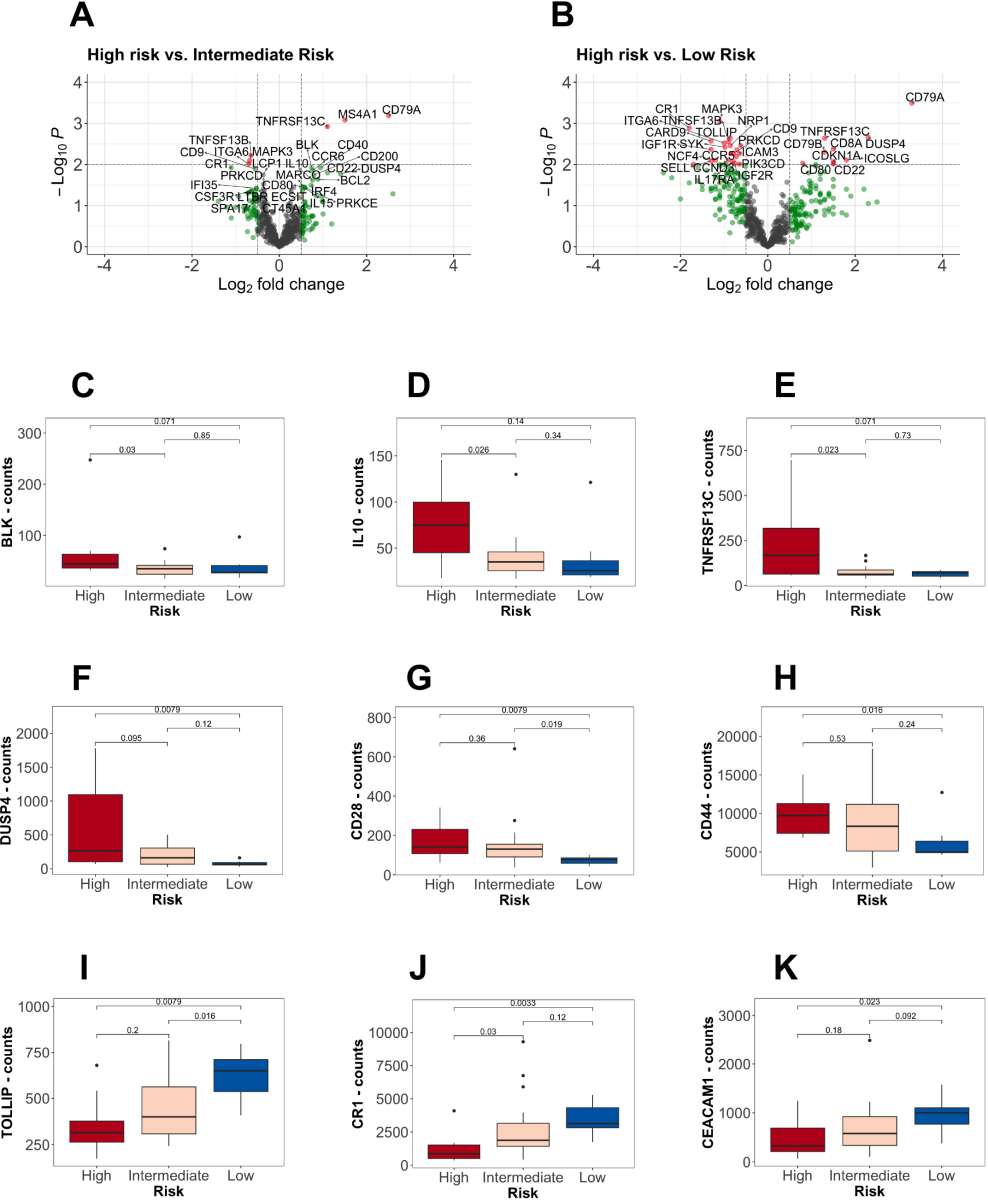
Differential gene expression analysis according to the risk stratification. **(A, B)** Volcano plots showing comparisons between high-risk versus (vs.) low and intermediate-risk. On the right (positive log-fold change values), the differential expressed genes upregulated in high-risk patients. In red, the genes with a p-value lower than 0.01 and a fold change greater than 0.5. **(C-H)** B-cell markers upregulated in high-risk patients. **(I-K)** Upregulation of specific markers in low-risk patients.

In total, 110 genes were differentially expressed between high and low risk and 4 genes between high and intermediate risk (**Figures 4A, B**). The most significant were B-cell related genes, given the increase in the B-cell (mean 9.0%, range 0 to 42.1%) and plasma-cell (mean 2.1%, range 0 to 10%) compartments inferred from gene expression. Only the plasma cells were positively correlated between gene expression and MFC (r=0.40; p=0.034) (**Supplementary Figure 2F**). Genes that showed an increasing gene expression pattern from low towards high-risk groups were BLK, TNFRSF13C, DUSP4, CD28, IL10 and CD44. On the contrary, TOLLIP, CEACAM1 and CR1 were upregulated in low-risk patients (**Figures 4C-K**). Other molecules of interest such as the IGF1R and IGF2R were downregulated in the high risk compared to the low-risk group. The expression of the immune checkpoints CTLA4, TIGIT, LAG3, PD1 and HAVCR2 did not show any differences (**Supplementary Figures 3A-E**). Considering the possibility of capturing gene expression signatures associated with the diagnosis rather than the risk categories, we further performed DGE analysis based on diagnosis. We did not find significant differences, except for the upregulation of ITGA6 in IgM MGUS compared to WM (log2FC 0.85, p<0.001, q-value=0.063), that was also observed in low-risk patients in contrast to high risk (log2FC 1.00, p=0.003, q-value=0.17). No differential expressed genes were found according to *MYD88* L265P or *CXCR4*, *KMT2D* and *ARID1A* mutations.

### Gene expression signatures of high and low tumor burden patients

**Figure 5:**
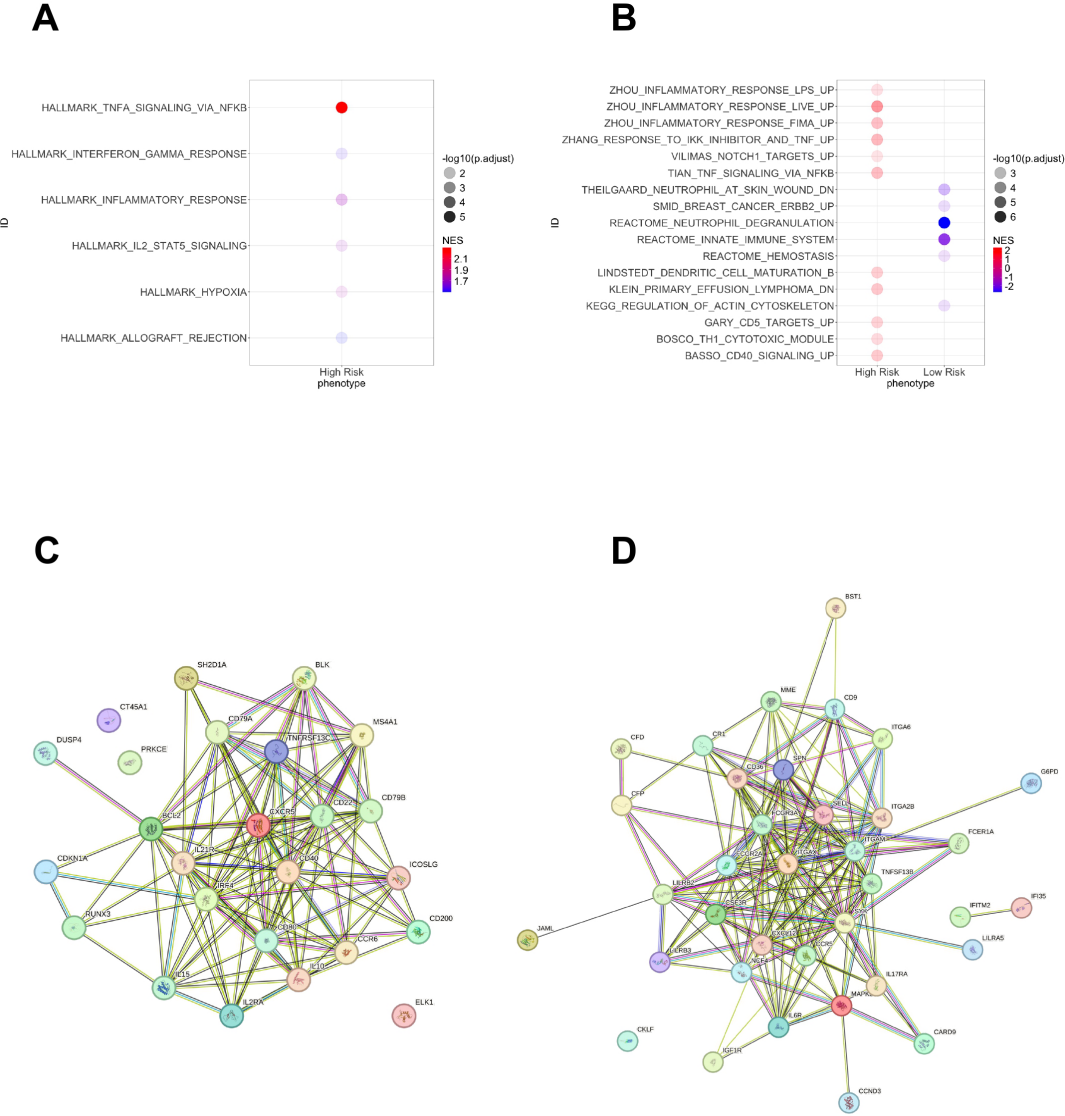
Gene set enrichment analysis in patients with high-risk IgM gammopathy and protein interaction networks. Intermediate and low risk were grouped as “low” risk patients to increase statistical power. All analyses used the Human Molecular Signatures Database. **(A)** Hallmark gene sets characteristic only in the high-risk group. **(B)** Canonical pathways gene sets comparing high and low risk. On the right of A and B are displayed the normalized enrichment score (NES). **(C)** Top upregulated genes in high-risk patients compared to intermediate and low risk. **(D)** Top upregulated genes in low-risk patients compared to high and intermediate risk.

**Figure 6:**
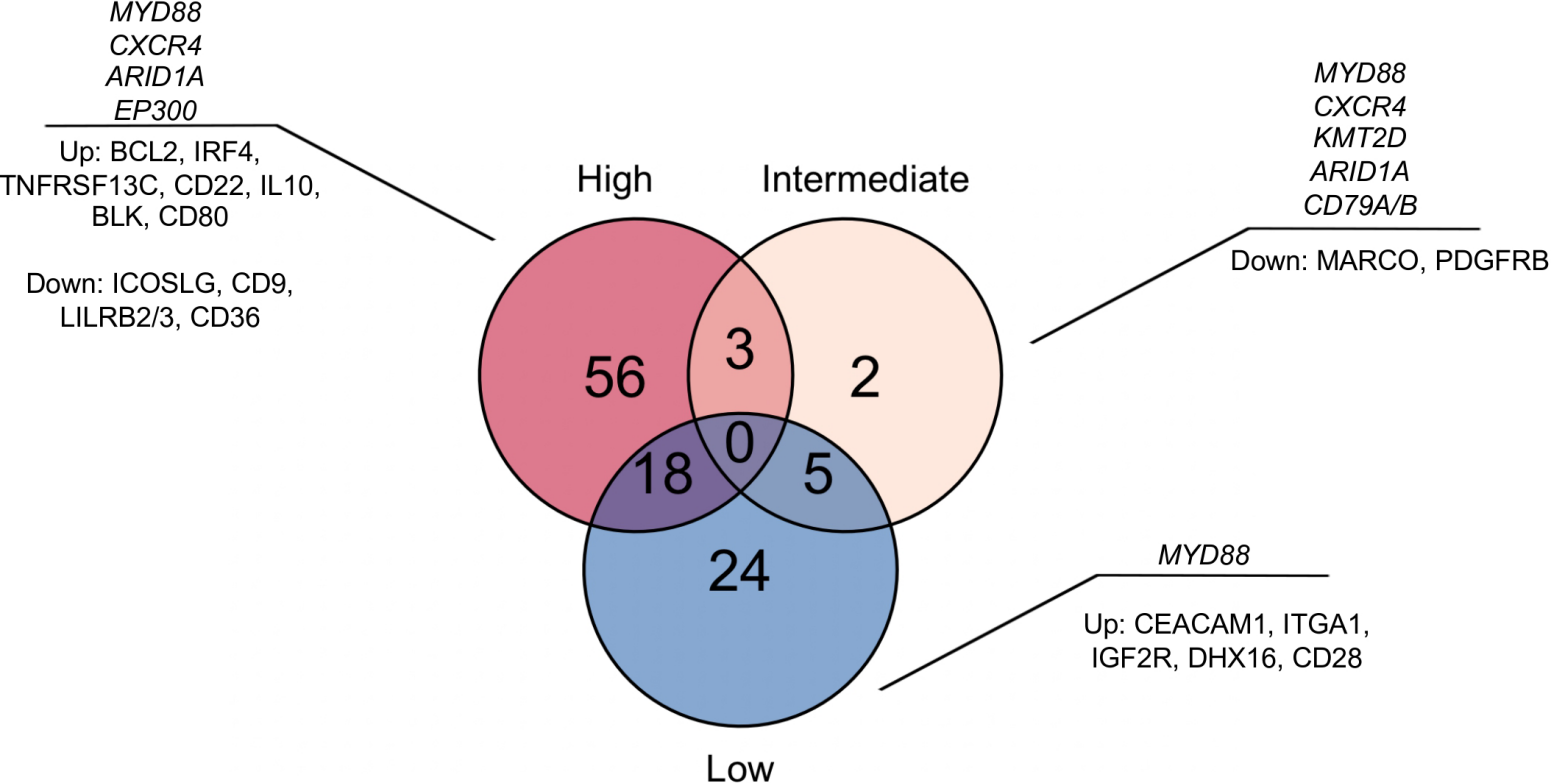
Model of tumor and immune microenvironment signatures for each risk category. The Venn diagram displays the upregulated unique genes in each risk category. The most frequent somatic mutations in driver genes (in italics) and top representative upregulated genes are shown.

We then performed enrichment analysis by merging low- and intermediate-risks into a single group, in contrast to high-risk. We observed that high-risk patients had a significant association with the NF-κB signaling and an inflammatory response mediated by IFN-gamma and the IL-2 mediated STAT5 activation (**Figure 5A**). Other signatures associated with the high-risk group were related to an inflammatory response mediated by the NF-κB and an activation of Th1 cytotoxic cells. Meanwhile, the low-risk group was characterized by an increase of myeloid-related compartment signatures (**Figure 5B**), reflecting the increased proportion of monocytes and neutrophils. Using the top upregulated genes in high and low/intermediate risk groups to infer protein interactions, we found that the high-risk was associated with the BCR signaling and expression of CD22, the B cell-activating factor receptor (BAFFR or TNFRSF13C), and key components of the BCR (CD79A/B). The low and intermediate risk networks were characterized by B-cell activation based on SYK (CR1, Fc gamma receptor IIa/IIIa, LILRB2, CXCL12, IL6R) (**Figures 5C, D**). SYK and MAPK1 showed a continuous increase from high to low risk (**Supplementary Figures 4A, B**). Along with B cell markers, the intermediate and low risk groups had a strong association with CD9 and CD36 involved in macrophage phagocytosis and inflammation (23, 24). To find the unique differentially expressed genes across risk groups, we obtained the significant genes (p<0.05) specific for each category. High- and low-risk groups had a higher number of unique expressed genes compared to the intermediate group (**Figure 6**).

## Discussion

We characterized the genomic and immune landscape of a recent prognostic risk classification of asymptomatic IgM gammopathy patients, as we reasoned that it could give a view of new mechanisms of progression beyond the classic biomarkers (11). We did not include previously treated WM patients due to the high heterogeneity and clonal diversification that might contribute to selection of biomarkers of treatment response instead. Given the intention of characterizing asymptomatic patients at diagnosis and the high number of IgM MGUS cases, which represented nearly half of our study population, we chose a prognostic model that included both IgM MGUS and asymptomatic WM (11), in contrast to other risk models that focused primarily on asymptomatic WM (10). Accordingly, the patients from our study were better classified in the Spanish risk model, as only intermediate and high-risk patients experienced disease progression.

Previous studies focused on the differences according to diagnosis; however, there can be challenges when comparing IgM MGUS and WM, as they share the same cell-of-origin (4, 15). In fact, we found that some IgM MGUS patients had immunophenotypical characteristics in close relationship with WM. Clonal B cells with expression of CD22 and CD25 along with a small population of clonal plasma cells were observed in some IgM MGUS patients. Moreover, ddPCR detected the *MYD88* L265P in a high proportion of IgM MGUS patients, similar to recent reports (7, 25, 26). To note, we also observed the MYD88 mutation in IgM MGUS patients without a clonal B-cell/plasma-cell compartment in the BM, reinforcing the concept of the *MYD88* L265P as an early clonal event in IgM gammopathy (15, 16).

Beyond *MYD88* L265P, *CXCR4* mutations were exclusively identified in intermediate and high-risk groups. Among them, *CXCR4* c.1025C>A (COSV54010290) was previously reported to increase the risk of progression in asymptomatic IgM gammopathy patients (6, 7). Another reported *CXCR4* mutation in WM and other B cell neoplasms was c.1012C>T (COSV54010080) (13, 27, 28), although there is limited data on its predictive impact on progression. Frameshift mutations were also found in our intermediate and high-risk cohorts and comprised the majority of *CXCR4* mutations. A recent study suggested that *CXCR4* mutations are present only in phenotypically abnormal B cells that harbored *MYD88* L265P mutations, suggesting a potential role as driver of disease progression (15). Our results support this model, in which none of the low-risk patients harbored *CXCR4* mutations. Other mutations involving key driver genes in WM, such as *KTM2D*, *ARID1A* and *EP300*, followed a same pattern in intermediate and high-risk groups, and could be putative drivers of disease progression. For instance, a study reported that *ARID1A* mutations were associated with high BM disease burden and decreased hemoglobin values in WM patients (29, 30). In the case of *KMT2D*, only one study reported a prevalence of 5% in IgM MGUS and 24% in WM patients using a targeted approach (5). In our study, although *KMT2D* mutations were frequent in the intermediate risk, only one WM patient progressed with a VAF of 1.1%, and had other 6 mutations involving *ARID1A*, *EP300*, *GNA13* and *TNFAIP3*. These suggest that *KMT2D* mutations might not drive disease progression in IgM gammopathy, as they might be subclonal. We also identified mutations in EP300 with high VAF in patients who progressed, some previously reported in DLBCL and recognized as drivers of progression (20). Moreover, another study reported an *EP300* mutation in a case of transformed WM to plasmablastic lymphoma (31). However, up to 19% of plasmablastic lymphoma patients were reported to harbor *EP300* mutations without previous history of IgM gammopathy (32). *EP300* mutations are also prevalent in follicular lymphoma, and it has been postulated that *EP300*-mutant lymphoma cells have an abnormal germinal center transcriptional regulation (33). Altogether, our results suggest that early mutations in key epigenetic regulators such as *ARID1A*, *EP300* and *KMT2D* are present in pre-symptomatic patients with high risk of disease progression. Other recurrent mutations found in WM such as *CD79B* and *TNFAIP3* have been associated with a more aggressive phenotype and transformation to DLBCL (34, 35). While in our study patients with *CD79B* mutations had stable disease, two out of three patients who had *TNFAIP3* mutations progressed. Inactivation of *TNFAIP3* is driven by 6q deletion or mutations, and it has been associated to treatment initiation in WM (36, 37), and also reported to be present in pre-symptomatic stages such as IgM MGUS (38).

On the other hand, low allele frequency mutations (VAF 1% - 5%) involving *BIRC3*, *NOTCH2*, *BCL2*, *CIITA*, *KRAS*, *MEF2B* and *NFKBIE* were identified in IgM MGUS patients with stable disease, except for one case with *MEF2B* who progressed. Among them, *BIRC3*, *BCL2*, *MEF2B*, *NFKBIE* and *NOTCH2* were present in *MYD88* wt patients, and might have conferred a survival advantage in small clones. For instance, a study reported *NOTCH2* mutations in stable and *MYD88* wt pretreatment WM (39). Despite *BIRC3* mutations were recently identified in high risk WM patients, we were not able to associate *BIRC3* mutations to a high-risk clinical phenotype due to its only presence in IgM MGUS with low mutation burden and no disease progression (40). Another recent study did not detect mutations in *BIRC3*, *NOTCH2* and *NFKBIE* in IgM MGUS or WM because their NGS assay did not cover these genomic regions (41). To what extent these mutations can further drive disease progression is yet to be investigated with larger cohorts and longer follow-up, especially in IgM MGUS patients.

Regarding the immune landscape, the low-risk group was characterized by higher proportion of myeloid cells, especially monocytes and neutrophils, and granulocytic related signatures. In line with our results, a study reported an early myeloid inflammation state in IgM MGUS and a fewer number of monocytes in WM using single cell protein screening and single cell RNA sequencing, which might contribute to a low response to interferon signaling and a compromised immune surveillance against the tumor (16, 19). More recently, a study demonstrated the expansion of pro-tumor monocytes and how they decreased after exposure to a vaccine in a clinical trial for WM (42). Given its easy applicability and previous results in multiple myeloma and other non-Hodgkin lymphomas, further studies are needed to confirm the impact of the monocytes counts in peripheral blood on disease progression (43, 44). On the opposite, activated mast cells had an increased trend towards high-risk, findings that were previously associated with higher risk of progression in WM (45).

Genes related to the BCR signaling pathway were upregulated in high-risk patients, such as BLK, CD79A, IRF4, BCL2, IL-10 and CD44. To note, we reported that TNFRSF13C (BAFFR) was upregulated in the high-risk group, an antigen that has shown promising results as a chimeric antigen receptor (CAR) target in CD19 negative B cell malignancies (46). CD44, involved in hematopoietic cell migration, was previously associated to be overexpressed in WM and IgM MGUS compared to healthy controls, but also in *MYD88* L265P activated B-cell (ABC) DLBCL (47). In contrast, the low-risk group upregulated CEACAM1, TOLLIP and CR1, that are associated with a BCR signaling blockade or an alternative activation of B cells (48–50). TOLLIP (Toll interacting protein) is an intrinsic modulator of the Toll-like receptor that controls the NF-kB activation mediated by MyD88 (51). Similarly, gene set enrichment analyses identified signatures related to increased cell junctions to neighboring cells, suggesting a localized stage of the disease in low-risk patients. Although we could not find differences regarding the proportion of macrophages across the risk categories, we found overexpression of CD9, CD36 and LILRB2 in intermediate- and low-risk groups, which can translate an early metabolic alteration and altered phagocytosis, as it was previously reported the interaction of CD9 and CD36 on macrophage surface (23, 24).

Still, intermediate- and low-risk patients upregulated SYK, MAPK1, IGF1R and IGF2R, suggesting early proliferative B cells, which is in line with the lower amount of clonal B cells in these groups. Specific signatures related to intermediate-risk group was more difficult to assess. Although multiple mutations were also identified in the latter group, the clinical outcome for the majority of these patients were similar to the low-risk. We highlight that the intermediate group is likely a transition state, similar to a study that reported an intermediate cluster characterized by the coexistence of inflammation and tumor cell proliferation (52). Altogether, high risk patients are characterized by an increased association with the BCR signaling pathway and upregulation of BCL2, and low-risk patients displayed an early immune myeloid deregulation, while trying to down regulate the activation of the NF-kB canonical BCR signaling.

A limitation of our study was related to the follow-up time and the limited number of patients included, as it could biased the identification of a very high-risk group. On the other hand, we did not identify differences in immune checkpoints expression across risk categories, probably due to the few T-cell subpopulations in the TME in our study. However, previous studies reported only a modest expression of PD-L1 in tumor cells and PD1 in T cells in WM samples (16, 53). In addition, there is now great focus on how aberrant myeloid cells change the TME to promote tumor progression by limiting the activation of cytotoxic T cells in WM (16, 42, 54). Another limitation is that the targeted gene expression panel used in our study allowed the identification of the most representative immune cell types. Still, this approach has been previously used by others in DLBCL with high accuracy (22). Regarding the DNA sequencing, we were limited to isolate CD138+ cells due to the very low tumor burden observed in IgM MGUS. However, the high depth of the targeted design was able to identify single nucleotide variants and putative drivers of progression with high sensitivity and confidence. Further validation of our results is needed given the high heterogeneity of disease progression.

In summary, we defined the genomic and immune landscape of the risk categories in IgM gammopathy. The low-risk group is characterized by the solely presence of the *MYD88* mutation, alternative activation of B cells, early myeloid deregulation and high monocytic compartment. In contrast, the high-risk group had *CXCR4*, *ARID1A* and *EP300* somatic mutations along with a strong association with the BCR activation that might further drive disease progression.

## Data Availability

The datasets generated for this study can be found in the GSE284434 in the Gene Expression Omnibus (https://www.ncbi.nlm.nih.gov/geo/query/acc.cgi?acc=GSE284434). All other information is available upon reasonable request to the corresponding authors.
