# Supplementary material for: Genomic and immune profiling of prognostic risk groups in IgM gammopathy reveals novel biomarkers beyond MYD88 L265P
Source: Front Immunol. 2025 Jul 1;16:1604089. doi: 10.3389/fimmu.2025.1604089 (PMC12259550; doi:10.3389/fimmu.2025.1604089)
Supplement: Supplementary file 1 [file DataSheet1.docx]

Supplementary Material

# Supplementary Data

Sample processing

BM samples were obtained from aspirate at the time of diagnosis and collected in EDTA tubes. B-cell clonality was assessed by multiparameter flow cytometry (MFC) using a BD FACSCanto II and analysed using FACSDiva software (BD Biosciences, San Jose, CA, USA) (Supplementary Table 1). ddPCR was used to detect the *MYD88* L265P mutation using a commercial assay (ID dHsaMDS2516944, Bio-Rad, Hercules, CA, USA). Droplets were generated on a Droplet Generator QX200 (Bio-Rad). The PCR product of each well was analyzed using the QX200 Droplet Reader (Bio-Rad). Data were analyzed with QuantaSoft Software 1.0 (Bio-Rad). The assay was able to detect a minimum of 0.025% of mutation burden, as previously described^1^. All samples were tested in duplicates, and only those containing at least 3 mutated droplets were informed as positive for the mutation.

Isolation of CD19+ cells was performed with using immunomagnetic microbeads on an AutoMACS instrument (Milteny Biotec, Bergisch Gladbach, Germany). The CD19+ enriched cells were washed and centrifuged to obtain a minimum of 95% purity. DNA isolation from CD19+ B cells was performed using the DNeasy® Kit (Qiagen, Hilden, Germany). The isolated DNA was then used for quantity and quality analyses using the Qubit High Sensitivity dsDNA kit (Thermo Fisher Scientific, Waltham, MA USA) and TapeStation (Agilent Technologies, Santa Clara, CA, USA), respectively. Total RNA was isolated from whole BM samples using TRIzol® reagent (Thermo Fisher). The isolated RNA was quantified using Qubit 2.0 Fluorometer (Thermo Fischer).

Identification of somatic mutations

FASTQ files were aligned to the reference human genome (GRCh38.p13) using BWA-MEM (version [v.] 0.7.17). The BAM files were sorted and indexed using samtools (v. 1.9). The data processing followed the best practices from the Genome Analysis Toolkit (GATK, v. 4.1.8.1). In short, PCR duplicates were marked using MarkDuplicates. Base quality score recalibration was performed using BaseRecalibrator and ApplyBQSR. Mutect2, Pisces (v. 5.3.0.0), VarScan2 (v. 2.3.9), and LoFreq (v. 2.1.5) were used to call variants (single nucleotide variants and small indels). Variants were normalized using bcftools (v. 1.10.2) and only those detected by at least 2 callers were used in downstream analyses to keep high confident variants, as described previously^2^. Variants with less than 1% of variant allele frequency (VAF) were excluded to minimize noise and ensure high-confidence mutation calls. SnpEff/SnpSift (v. 5.1d) was used to annotate variants. Variants reported in the 1000 Genomes Project, ExAC or gnomAD with a population frequency greater than 1% were reported as germline. Polyphen and SIFT were used for further prediction of putative damaging variants.

Gene expression analysis

Quality control of the raw data followed the recommendations of the nSolver software. The R package NanoTube (v. 1.10.0) with standard parameters was used to preprocess the gene expression counts. In short, the geometric mean of the housekeeping genes was used to normalize the gene counts between samples^3^. Limma (v. 3.60.6) was used for differential expression analyses, and p-values were adjusted using the Benjamini Hochberg method. Gene set enrichment analysis (GSEA) was performed on the set of filtered gene counts. Msigdbr (v. 7.5.1) and clusterProfiler (v. 4.12.6) were used to obtain enrichment scores from the MSigDB (Molecular Signature Database). The p-value cutoff less than 0.01 was used to find enriched gene sets. Protein to protein interactions were assessed in STRING (v12.0) with high confidence (>0.70) as the minimal interaction score. To better characterize patients that have progressed during follow-up and that might display a more aggressive phenotype, we considered low-risk to both intermediate- and low-risk groups in the gene set enrichment analyses. Cell-types imputation was performed using CIBERSORTx with the LM22 signature matrix as reference^4^.

**References**

1. Moreno DF, López‐Guerra M, Paz S, et al. Prognostic impact of MYD88 and CXCR4 mutations assessed by droplet digital polymerase chain reaction in IGM monoclonal gammopathy of undetermined significance and smouldering Waldenström macroglobulinaemia. Br J Haematol. 2023;200(2):187–196.

2. Nadeu F, Royo R, Massoni-Badosa R, et al. Detection of early seeding of Richter transformation in chronic lymphocytic leukemia. Nat. Med. 2022;28(8):1662–1671.

3. Class CA, Lukan CJ, Bristow CA, Do K-A. Easy NanoString nCounter data analysis with the NanoTube. Bioinformatics. 2023;39(1):btac762.

4. Newman AM, Liu CL, Green MR, et al. Robust enumeration of cell subsets from tissue expression profiles. Nat Methods. 2015;12(5):453–457.

# Supplementary Figures and Tables

## Supplementary Tables

**Supplementary Table 1.** Multiparametric flow cytometry markers

|  | **Tube 1** | **Tube 2** | **Tube 3** |
| --- | --- | --- | --- |
| FITC | CD8+IgL | CD23 | FMC7 |
| PE | CD56+IgK | CD10 | CD25 |
| PerCPCy5-5 | CD56+IgK | CD79b | CD11c |
| PC7 | CD19+TCR | CD19 | CD19 |
| APC | CD3 | CD200 | CD22 |
| APC H7 | CD38 | CD43 | IgL |
| V450 | CD20+CD4 | CD200 | IgK |
| V500 | CD45 | CD45 | CD45 |

**Supplementary Table 2.** Panel of genes covered in the DNA sequencing analysis

| *ARID1A* | *CREBBP* | *NOTCH2* |
| --- | --- | --- |
| *ATM* | *CXCR4* | *NRAS* |
| *B2M* | *EP300* | *PAX5* |
| *BCL2* | *EZH2* | *PIM1* |
| *BCL6* | *FBXW7* | *PLCG2* |
| *BIRC* | *FOXO1* | *POT1* |
| *BRAF* | *GNA13* | *PRDM1* |
| *BTK* | *ID3* | *PTEN* |
| *CARD11* | *IRF4* | *PTPN11* |
| *CCND1* | *KMT2A* | *REL* |
| *CCND3* | *KMT2D* | *SF3B1* |
| *CD58* | *KRAS* | *SOCS1* |
| *CD79A* | *MAL* | *STAT6* |
| *CD79B* | *MEF2B* | *TCF3* |
| *CDKN2A* | *MYC* | *TNFAIP3* |
| *CDKN2B* | *MYD88* | *TNFRSF14* |
| *CHD2* | *NFKBIE* | *TP53* |
| *CIITA* | *NOTCH1* | *XPO1* |

**Supplementary Table 3.** Somatic mutations in all patients. ID: identification; VAF: variant allele frequency.

| ID | Diagnosis | Risk | Outcome | Chromosome | Position | Reference allele | Alternate allele | Gene | Protein sequence | COSMIC ID | VAF |
| --- | --- | --- | --- | --- | --- | --- | --- | --- | --- | --- | --- |
| PNT1 | WM | High | Progression | 2 | 136114915 | G | T | *CXCR4* | p.Ser342* | COSV54010290 | 0.10 |
| PNT1 | WM | High | Progression | 22 | 41178506 | TCAG | T | *EP300* | p.Gln2267del | NA | 0.41 |
| PNT1 | WM | High | Progression | 3 | 38141150 | T | C | *MYD88* | p.Leu260Pro | COSV57169334 | 0.11 |
| PNT1 | WM | High | Progression | X | 101356177 | A | G | *BTK* | p.Cys515Arg | COSV58117643 | 0.10 |
| PNT1 | WM | High | Progression | X | 101356177 | A | T | *BTK* | p.Cys515Ser | COSV58117871 | 0.01 |
| PNT2 | WM | Intermediate | Stable | 17 | 63929439 | A | T | *CD79B* | p.Tyr197Asn,p.Tyr69Asn | COSV50076307 | 0.07 |
| PNT2 | WM | Intermediate | Stable | 2 | 136114915 | G | T | *CXCR4* | p.Ser342* | COSV54010290 | 0.05 |
| PNT2 | WM | Intermediate | Stable | 3 | 38141150 | T | C | *MYD88* | p.Leu260Pro | COSV57169334 | 0.07 |
| PNT28 | IgM MGUS | Intermediate | Stable | 1 | 119915811 | AT | A | *NOTCH2* | p.Ile2304fs | NA | 0.01 |
| PNT28 | IgM MGUS | Intermediate | Stable | 12 | 49034634 | G | A | *KMT2D* | p.Ser3463Leu | NA | 0.04 |
| PNT3 | WM | High | Stable | 2 | 136114901 | C | CA | *CXCR4* | p.Glu347fs | NA | 0.04 |
| PNT3 | WM | High | Stable | 2 | 136114917 | A | ATG | *CXCR4* | p.Ser342fs | NA | 0.03 |
| PNT3 | WM | High | Stable | 3 | 38141150 | T | C | *MYD88* | p.Leu260Pro | COSV57169334 | 0.33 |
| PNT3 | WM | High | Stable | 9 | 37033986 | C | T | *PAX5* | p.Gly16Arg | COSV63911668 | 0.37 |
| PNT5 | WM | High | Progression | 3 | 38141150 | T | C | *MYD88* | p.Leu260Pro | COSV57169334 | 0.43 |
| PNT6 | WM | Intermediate | Stable | 19 | 41880674 | G | C | *CD79A* | p.Asp250His | NA | 0.02 |
| PNT6 | WM | Intermediate | Stable | 2 | 136114928 | G | A | *CXCR4* | p.Arg338* | COSV54010080 | 0.40 |
| PNT6 | WM | Intermediate | Stable | 3 | 38141150 | T | C | *MYD88* | p.Leu260Pro | COSV57169334 | 0.37 |
| PNT29 | WM | High | Progression | 4 | 152328358 | C | A | *FBXW7* | p.Gly423Val | COSV55892390 | 0.01 |
| PNT26 | WM | High | Progression | 1 | 26774683 | C | T | *ARID1A* | p.Gln1486* | NA | 0.65 |
| PNT26 | WM | High | Progression | 1 | 116544389 | TTAAGTTGTAGA | T | *CD58* | p.Ile92fs | NA | 0.01 |
| PNT26 | WM | High | Progression | 2 | 136114928 | G | A | *CXCR4* | p.Arg338* | COSV54010080 | 0.36 |
| PNT26 | WM | High | Progression | 3 | 38141150 | T | C | *MYD88* | p.Leu260Pro | COSV57169334 | 0.66 |
| PNT7 | IgM MGUS | Intermediate | Stable | 22 | 41146776 | T | G | *EP300* | p.Ser697Arg | COSV54331593 | 0.50 |
| PNT7 | IgM MGUS | Intermediate | Stable | 3 | 38141150 | T | C | *MYD88* | p.Leu260Pro | COSV57169334 | 0.13 |
| PNT8 | WM | Intermediate | Stable | 12 | 49039963 | C | A | *KMT2D* | p.Glu2603* | COSV105895546 | 0.29 |
| PNT8 | WM | Intermediate | Stable | 3 | 38141150 | T | C | *MYD88* | p.Leu260Pro | COSV57169334 | 0.27 |
| PNT9 | IgM MGUS | Intermediate | Stable | 12 | 49034911 | T | C | *KMT2D* | p.Asp3419Gly | COSV56434929 | 0.48 |
| PNT9 | IgM MGUS | Intermediate | Stable | 16 | 10895742 | C | CAG | *CIITA* | p.Glu93fs | NA | 0.01 |
| PNT9 | IgM MGUS | Intermediate | Stable | 17 | 63929438 | T | A | *CD79B* | p.Tyr197Phe,p.Tyr69Phe | COSV50076492 | 0.01 |
| PNT9 | IgM MGUS | Intermediate | Stable | 3 | 38141150 | T | C | *MYD88* | p.Leu260Pro | COSV57169334 | 0.04 |
| PNT10 | WM | Low | Stable | 3 | 38141150 | T | C | *MYD88* | p.Leu260Pro | COSV57169334 | 0.30 |
| PNT11 | IgM MGUS | Low | Stable | 3 | 38141150 | T | C | *MYD88* | p.Leu260Pro | COSV57169334 | 0.18 |
| PNT12 | WM | Intermediate | Stable | 11 | 118468830 | C | G | *KMT2A* | p.Thr163Arg,. | NA | 0.48 |
| PNT12 | WM | Intermediate | Stable | 3 | 38141150 | T | C | *MYD88* | p.Leu260Pro | COSV57169334 | 0.31 |
| PNT30 | WM | Intermediate | Progression | 1 | 26731539 | C | A | *ARID1A* | p.Pro580Thr | NA | 0.01 |
| PNT30 | WM | Intermediate | Progression | 1 | 26732787 | T | G | *ARID1A* | p.Leu639Val | NA | 0.19 |
| PNT30 | WM | Intermediate | Progression | 12 | 49048002 | C | T | *KMT2D* | p.Cys1400Tyr | NA | 0.01 |
| PNT30 | WM | Intermediate | Progression | 17 | 65014258 | C | T | *GNA13* | p.Ter378Ter | NA | 0.50 |
| PNT30 | WM | Intermediate | Progression | 22 | 41146776 | T | G | *EP300* | p.Ser697Arg | COSV54331593 | 0.44 |
| PNT30 | WM | Intermediate | Progression | 22 | 41170438 | C | T | *EP300* | p.Pro1440Leu | COSV54329411 | 0.19 |
| PNT30 | WM | Intermediate | Progression | 6 | 137874967 | TTCCGCTGGC | T | *TNFAIP3* | p.Phe140_Gln143delinsTer | NA | 0.21 |
| PNT13 | IgM MGUS | Intermediate | Stable | 17 | 65014730 | C | G | *GNA13* | p.Val221Leu | COSV71474917 | 0.46 |
| PNT13 | IgM MGUS | Intermediate | Stable | 3 | 38141150 | T | C | *MYD88* | p.Leu260Pro | COSV57169334 | 0.04 |
| PNT14 | IgM MGUS | Low | Stable | 3 | 38141150 | T | C | *MYD88* | p.Leu260Pro | COSV57169334 | 0.07 |
| PNT31 | IgM MGUS | Low | Stable | 11 | 102337029 | G | A | *BIRC3* | p.Cys622Tyr | COSV54815152 | 0.02 |
| PNT31 | IgM MGUS | Low | Stable | 12 | 49048722 | ATCT | A | *KMT2D* | p.Glu1355del | NA | 0.01 |
| PNT31 | IgM MGUS | Low | Stable | 6 | 44265594 | G | GC | *NFKBIE* | p.Ala57fs | NA | 0.01 |
| PNT32 | WM | High | Progression | 16 | 3731781 | T | C | *CREBBP* | p.Lys1629Glu | NA | 0.01 |
| PNT32 | WM | High | Progression | 2 | 136114911 | AGATGAATGTCCACCTCG | A | *CXCR4* | p.Arg338fs | NA | 0.01 |
| PNT32 | WM | High | Progression | 22 | 41150154 | C | A | *EP300* | p.Pro925Thr | COSV54339374 | 0.16 |
| PNT32 | WM | High | Progression | 3 | 38141150 | T | C | *MYD88* | p.Leu260Pro | COSV57169334 | 0.01 |
| PNT32 | WM | High | Progression | 8 | 127738358 | C | CCAG | *MYC* | p.Gln52dup | NA | 0.06 |
| PNT32 | WM | High | Progression | 9 | 37006497 | C | T | *PAX5* | p.Val151Ile | COSV63906012 | 0.03 |
| PNT33 | WM | Low | Stable | 3 | 38141150 | T | C | *MYD88* | p.Leu260Pro | COSV57169334 | 0.16 |
| PNT16 | IgM MGUS | Intermediate | Stable | 11 | 118504857 | G | A | *KMT2A* | p.Glu3013Lys | COSV63292808 | 0.47 |
| PNT16 | IgM MGUS | Intermediate | Stable | 2 | 136114917 | A | AT | *CXCR4* | p.His341fs | NA | 0.01 |
| PNT16 | IgM MGUS | Intermediate | Stable | 3 | 38141150 | T | C | *MYD88* | p.Leu260Pro | COSV57169334 | 0.03 |
| PNT17 | WM | Low | Stable | 3 | 38141150 | T | C | *MYD88* | p.Leu260Pro | COSV57169334 | 0.43 |
| PNT18 | IgM MGUS | High | Stable | 17 | 63929439 | A | C | *CD79B* | p.Tyr197Asp,p.Tyr69Asp | COSV50076112 | 0.01 |
| PNT18 | IgM MGUS | High | Stable | 22 | 41149147 | C | T | *EP300* | p.Pro784Leu | COSV54325945 | 0.51 |
| PNT18 | IgM MGUS | High | Stable | 3 | 38141150 | T | C | *MYD88* | p.Leu260Pro | COSV57169334 | 0.17 |
| PNT19 | IgM MGUS | Intermediate | Stable | 12 | 25245345 | C | T | *KRAS* | p.Val14Ile | COSV55501342 | 0.05 |
| PNT19 | IgM MGUS | Intermediate | Stable | 16 | 3731830 | GT | G | *CREBBP* | p.Asn1612fs | NA | 0.02 |
| PNT19 | IgM MGUS | Intermediate | Stable | 3 | 38141150 | T | C | *MYD88* | p.Leu260Pro | COSV57169334 | 0.18 |
| PNT34 | IgM MGUS | Intermediate | Stable | 3 | 38141150 | T | C | *MYD88* | p.Leu260Pro | COSV57169334 | 0.01 |
| PNT20 | IgM MGUS | Intermediate | Stable | 11 | 102336925 | AC | A | *BIRC3* | p.Gln588fs | COSV54815749 | 0.01 |
| PNT20 | IgM MGUS | Intermediate | Stable | 17 | 63929438 | T | C | *CD79B* | p.Tyr197Cys,p.Tyr69Cys | COSV50076054 | 0.01 |
| PNT20 | IgM MGUS | Intermediate | Stable | 17 | 63929438 | T | G | *CD79B* | p.Tyr197Ser,p.Tyr69Ser | COSV50076156 | 0.01 |
| PNT20 | IgM MGUS | Intermediate | Stable | 2 | 136114898 | ACT | A | *CXCR4* | p.Glu347fs | NA | 0.03 |
| PNT20 | IgM MGUS | Intermediate | Stable | 3 | 38141150 | T | C | *MYD88* | p.Leu260Pro | COSV57169334 | 0.12 |
| PNT21 | IgM MGUS | Intermediate | Stable | 12 | 49026791 | G | A | *KMT2D* | p.His5059Tyr | COSV106058803 | 0.01 |
| PNT21 | IgM MGUS | Intermediate | Stable | 2 | 136114924 | C | CCT | *CXCR4* | p.Gly339fs | NA | 0.01 |
| PNT21 | IgM MGUS | Intermediate | Stable | 3 | 38141150 | T | C | *MYD88* | p.Leu260Pro | COSV57169334 | 0.04 |
| PNT21 | IgM MGUS | Intermediate | Stable | 6 | 137874871 | A | G | *TNFAIP3* | p.Thr108Ala | COSV52799446 | 0.01 |
| PNT21 | IgM MGUS | Intermediate | Stable | 9 | 22008816 | G | T | *CDKN2B* | p.Phe46Leu | NA | 0.02 |
| PNT22 | WM | Intermediate | Stable | 1 | 26766292 | A | G | *ARID1A* | p.Asn935Ser | COSV105877729 | 0.01 |
| PNT22 | WM | Intermediate | Stable | 3 | 38141150 | T | C | *MYD88* | p.Leu260Pro | COSV57169334 | 0.29 |
| PNT22 | WM | Intermediate | Stable | 9 | 22008816 | G | T | *CDKN2B* | p.Phe46Leu | NA | 0.01 |
| PNT35 | IgM MGUS | High | Progression | 1 | 26766292 | A | G | *ARID1A* | p.Asn935Ser | COSV105877729 | 0.01 |
| PNT35 | IgM MGUS | High | Progression | 19 | 19146330 | A | G | *MEF2B* | p.Leu282Pro | NA | 0.01 |
| PNT35 | IgM MGUS | High | Progression | 6 | 137874871 | A | G | *TNFAIP3* | p.Thr108Ala | COSV52799446 | 0.02 |
| PNT35 | IgM MGUS | High | Progression | 9 | 22008816 | G | T | *CDKN2B* | p.Phe46Leu | NA | 0.06 |
| PNT24 | IgM MGUS | Intermediate | Stable | 2 | 136114915 | G | T | *CXCR4* | p.Ser342* | COSV54010290 | 0.01 |
| PNT24 | IgM MGUS | Intermediate | Stable | 3 | 38141150 | T | C | *MYD88* | p.Leu260Pro | COSV57169334 | 0.01 |
| PNT23 | WM | High | Stable | 1 | 26773821 | C | T | *ARID1A* | p.Gln1342* | COSV61381745 | 0.01 |
| PNT23 | WM | High | Stable | 12 | 49034297 | C | A | *KMT2D* | p.Glu3504* | NA | 0.01 |
| PNT23 | WM | High | Stable | 2 | 136114901 | CAGTGG | C | *CXCR4* | p.Ser345fs | NA | 0.01 |
| PNT23 | WM | High | Stable | 2 | 136114982 | C | CG | *CXCR4* | p.Ala320fs | NA | 0.07 |
| PNT23 | WM | High | Stable | 3 | 38141150 | T | C | *MYD88* | p.Leu260Pro | COSV57169334 | 0.37 |
| PNT25 | IgM MGUS | Low | Stable | 3 | 38141150 | T | C | *MYD88* | p.Leu260Pro | COSV57169334 | 0.05 |
| PNT27 | IgM MGUS | Intermediate | Progression | 3 | 38141150 | T | C | *MYD88* | p.Leu260Pro | COSV57169334 | 0.01 |
| PNT36 | IgM MGUS | Intermediate | Stable | 18 | 63318214 | A | C | *BCL2* | p.Phe151Leu | NA | 0.01 |

## Supplementary Figures

**Supplementary Figure 1.** A. Correlation between the allele frequency of the *MYD88* L265P detected by next generation sequencing (NGS) with the mutation burden assessed by droplet digital polymerase chain reaction (ddPCR). B. Frequency of the mutated genes according to diagnosis. MGUS: Monoclonal gammopathy of undetermined significance. WM: Waldenström macroglobulinemia. R: Pearson correlation coefficient.

**Supplementary Figure 2.** Scatterplots showing the correlation of multiparameter flow cytometry (MFC) data and inferred cell populations of T cells and NK cells from gene expression (A-C). Distribution of the immune cell types based on diagnosis (D) and based on the presence of the *MYD88* L265P mutation (E). Scatterplot showing the correlation between MFC plasma cells and inferred gene expression plasma cells (F). MGUS: Monoclonal gammopathy of undetermined significance.

**Supplementary Figure 3.** Gene expression of immune checkpoints based on risk categories (A-E).

**Supplementary Figure 4.** mRNA expression counts of SYK and MAPK1 based on risk categories (A-B).

**Supplementary Figure 1.**


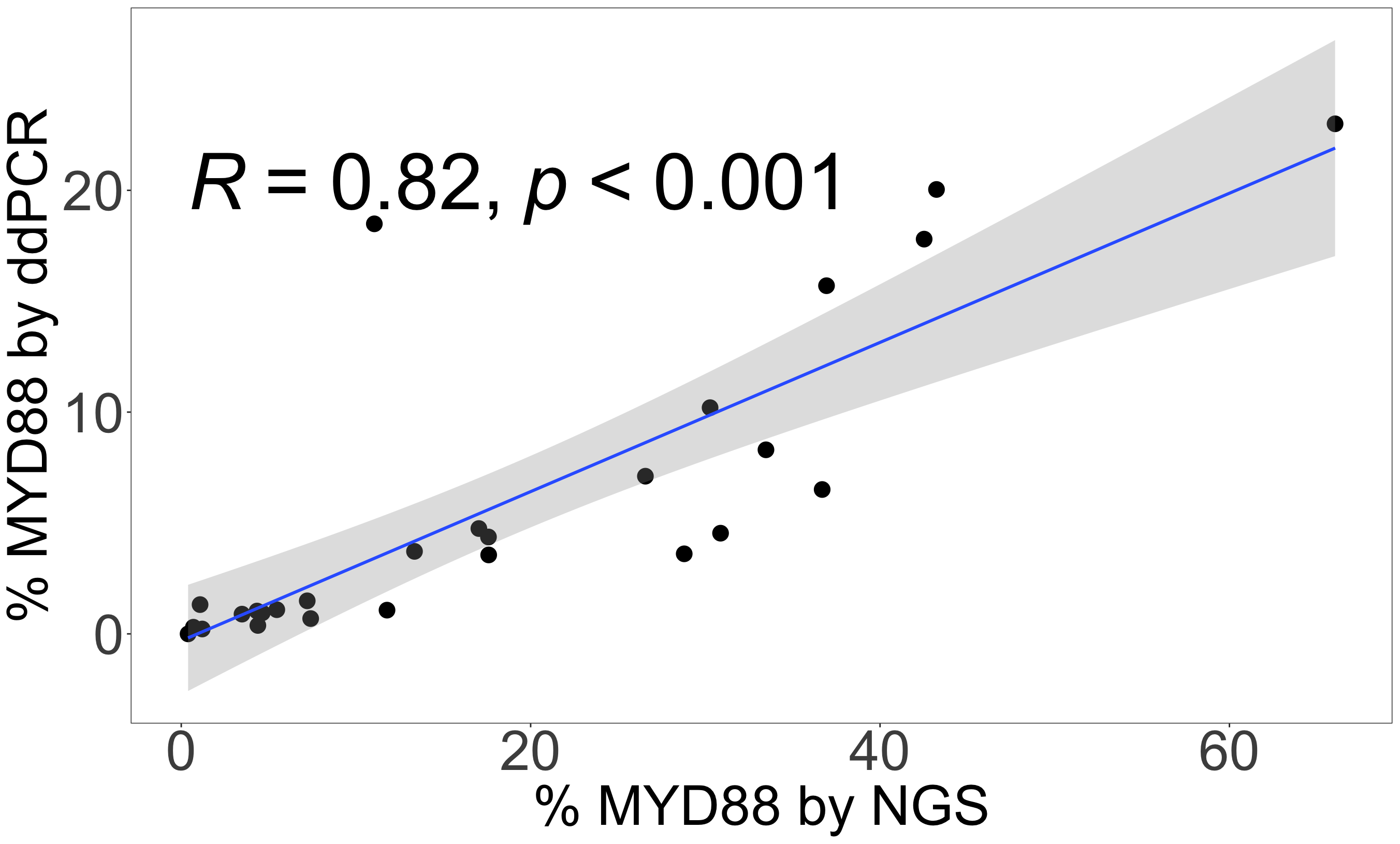

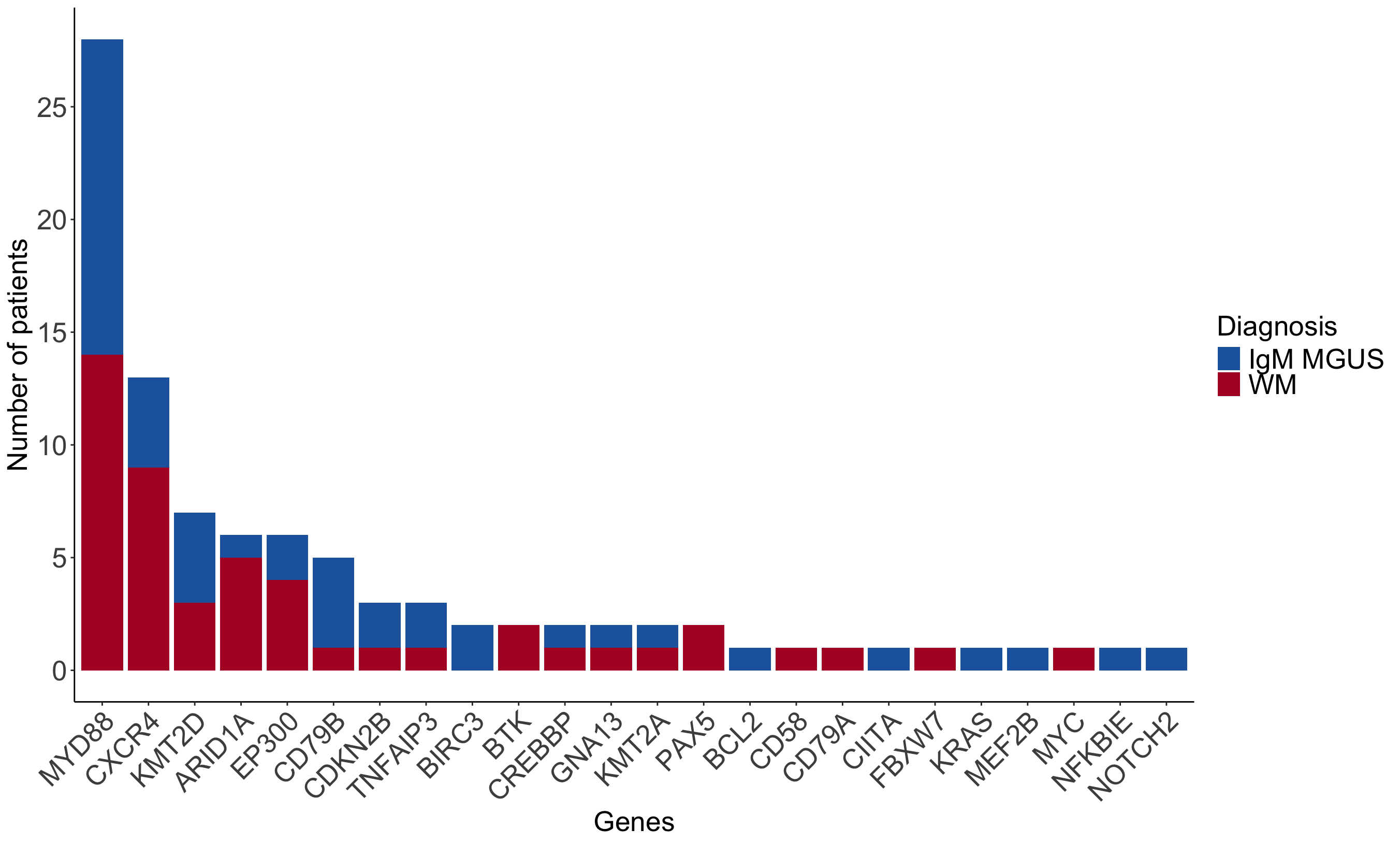


**A**

**B**

**Supplementary Figure 2.**

**B**

**C**

**A**

**
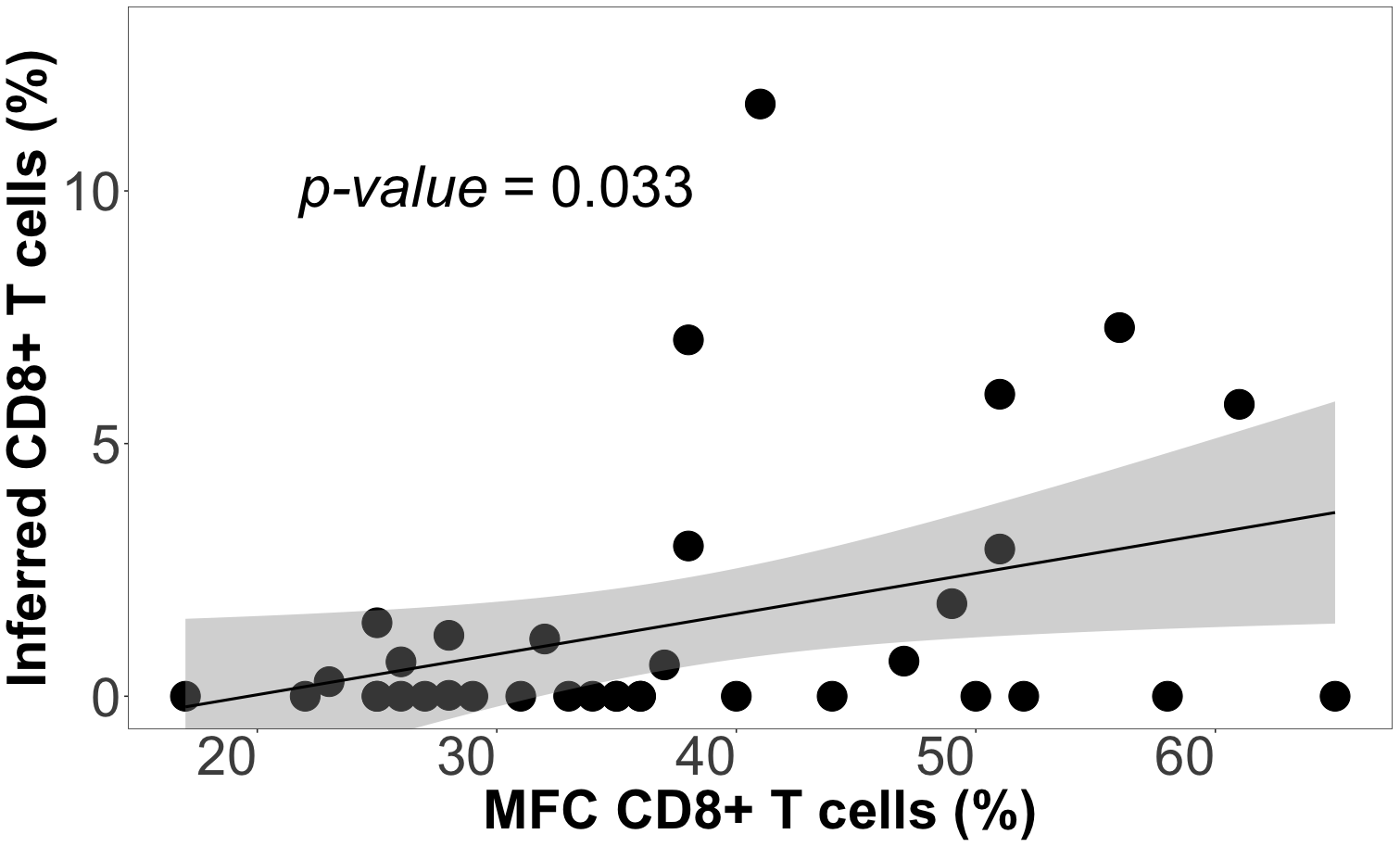

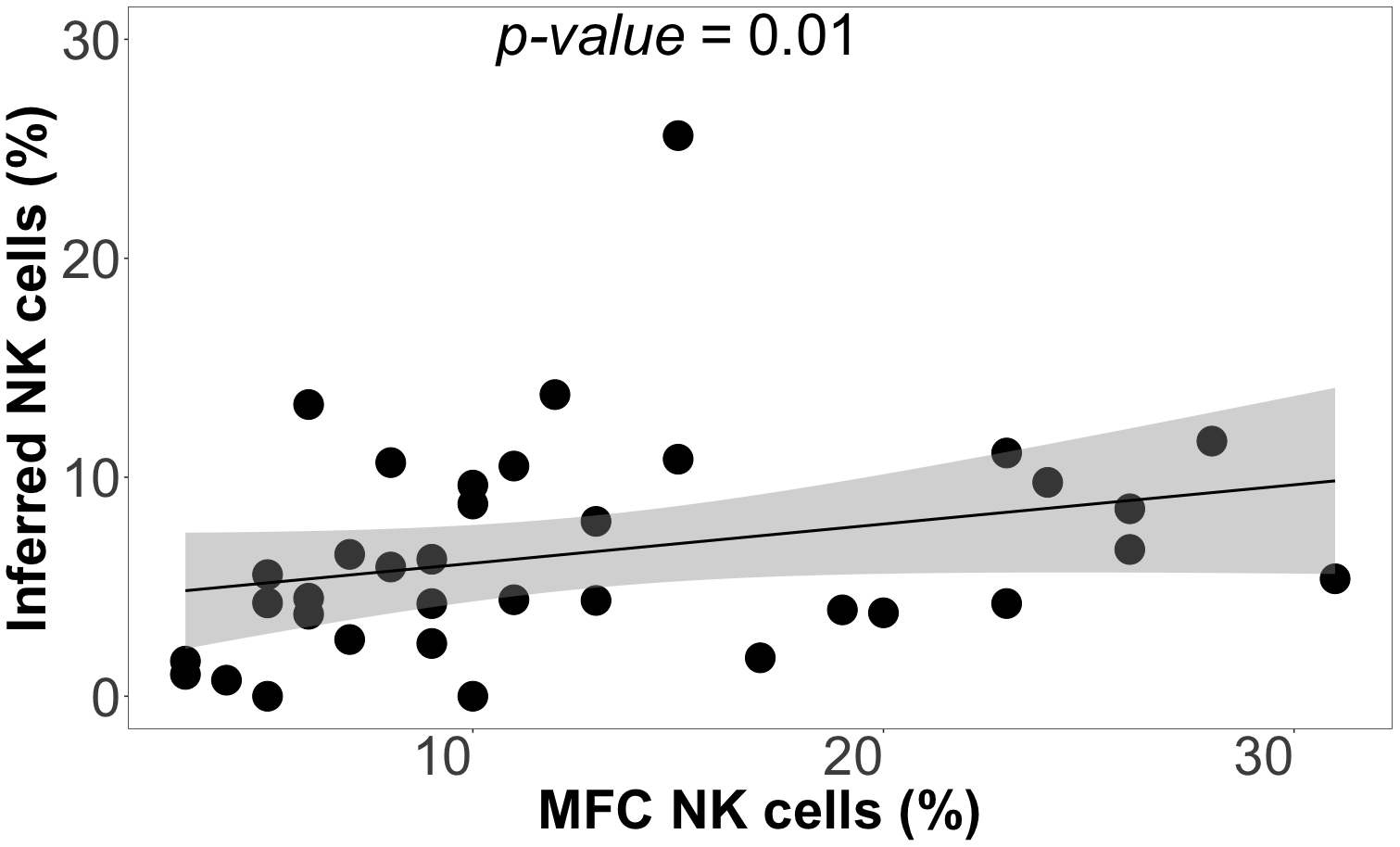

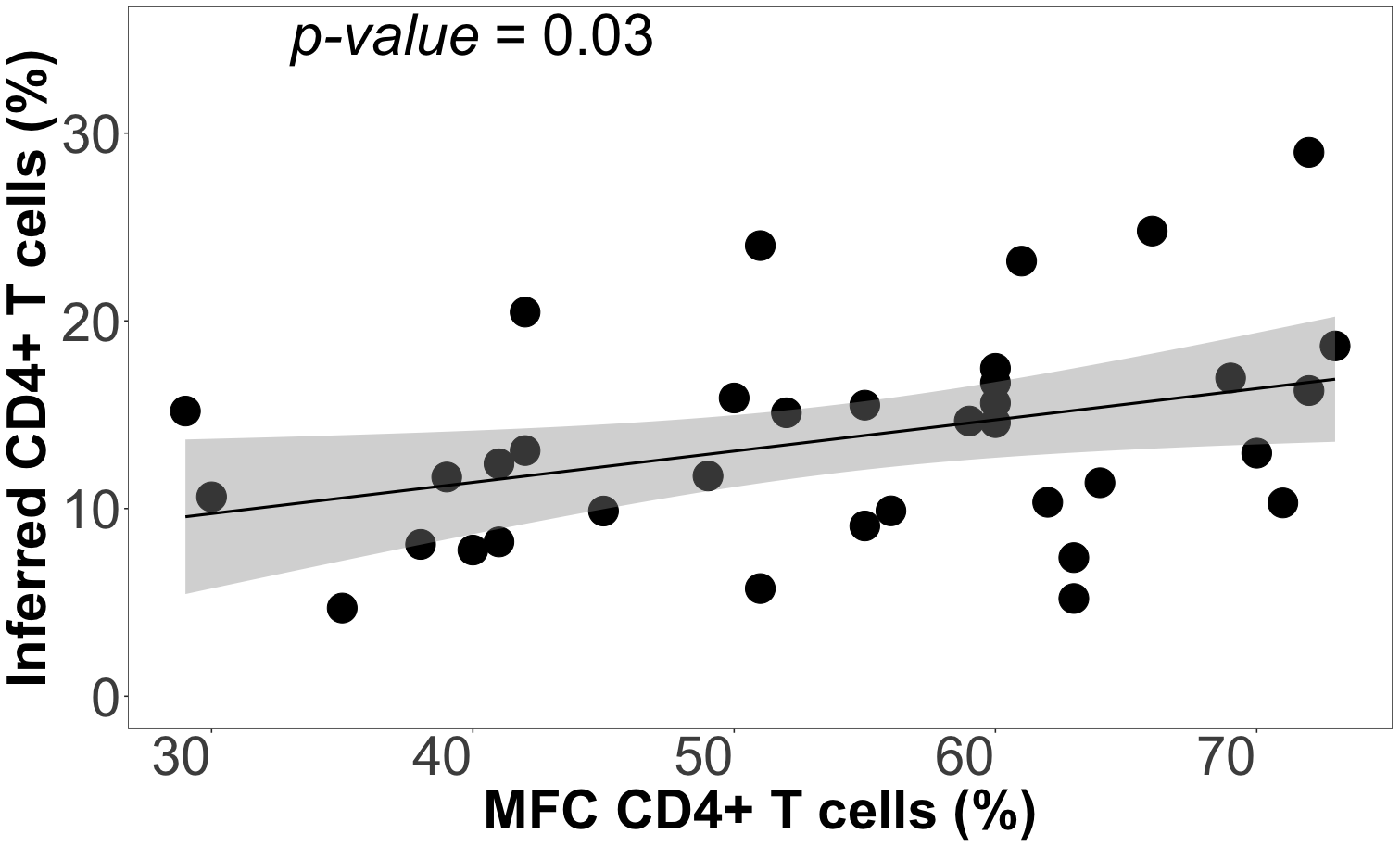
**

**D**

**
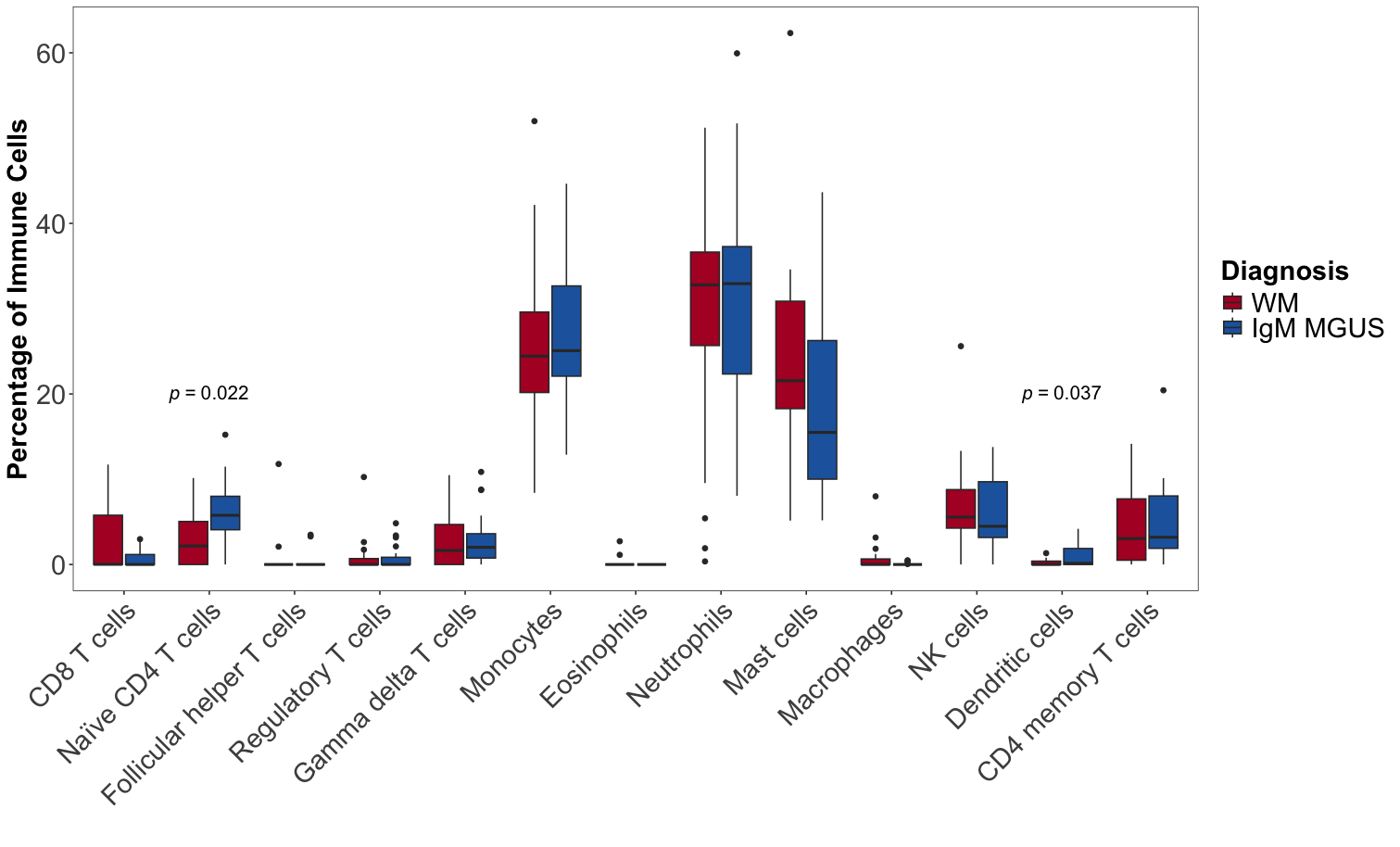

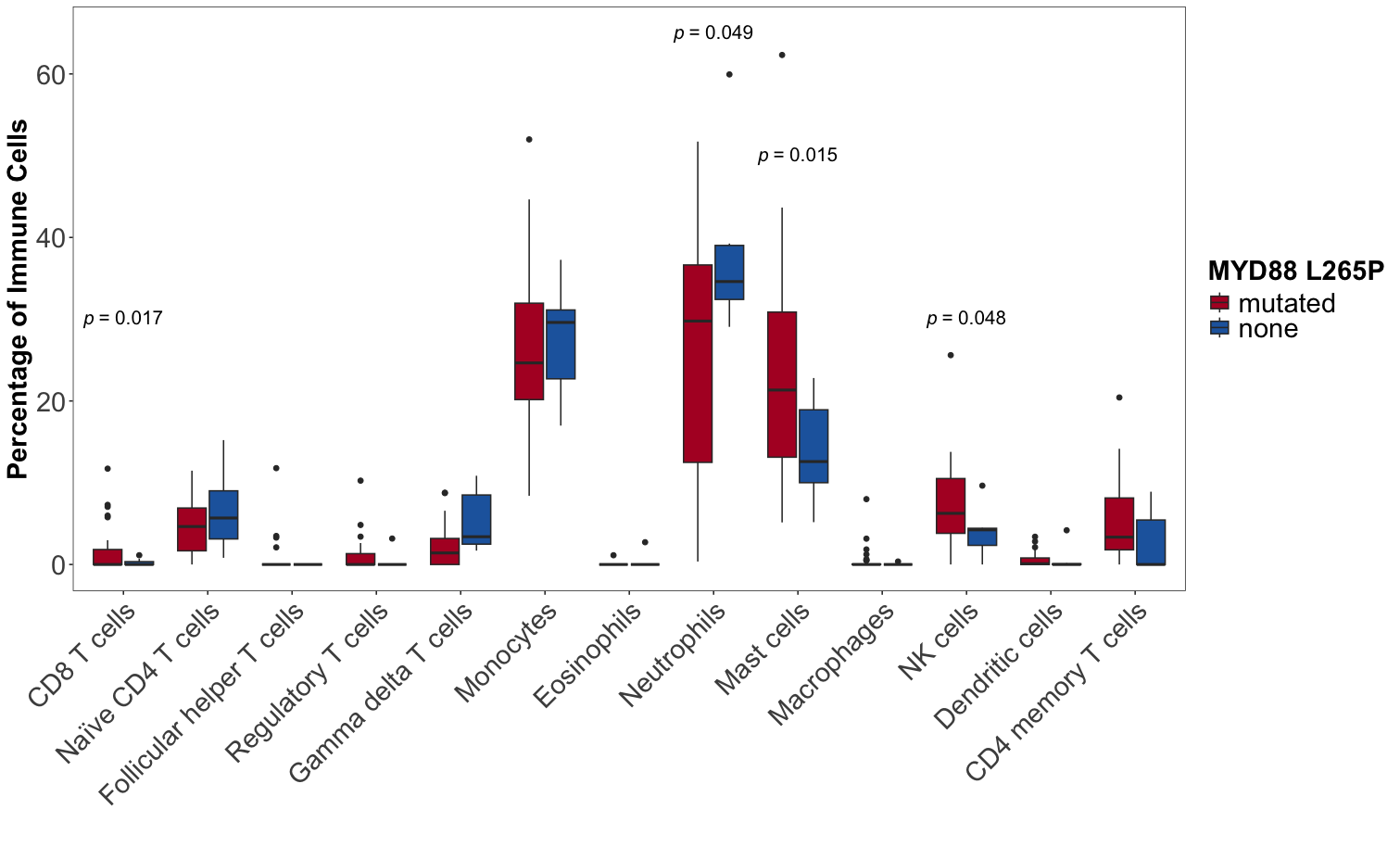
**

**E**

**F**

**
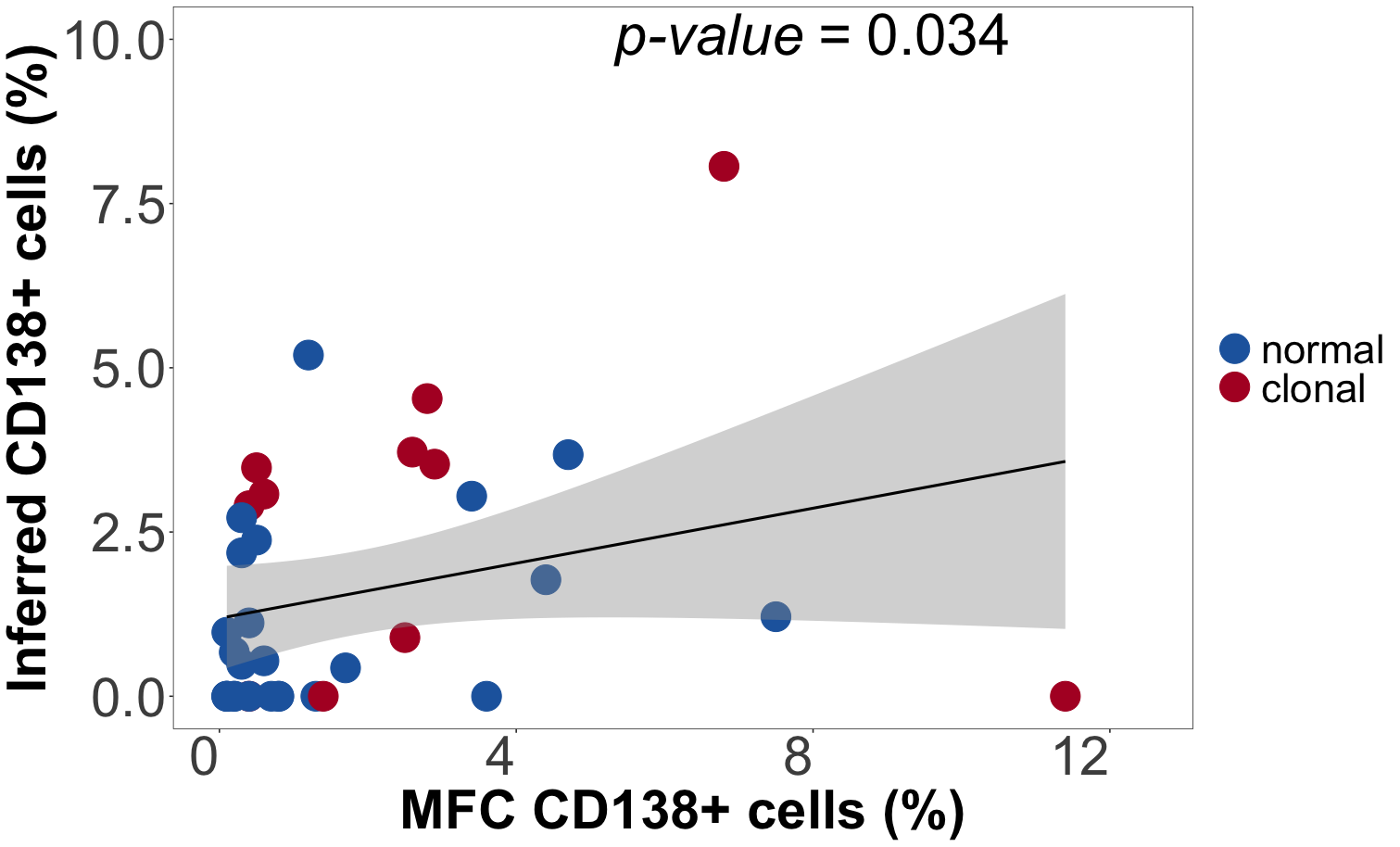
**

**Supplementary Figure 3.**


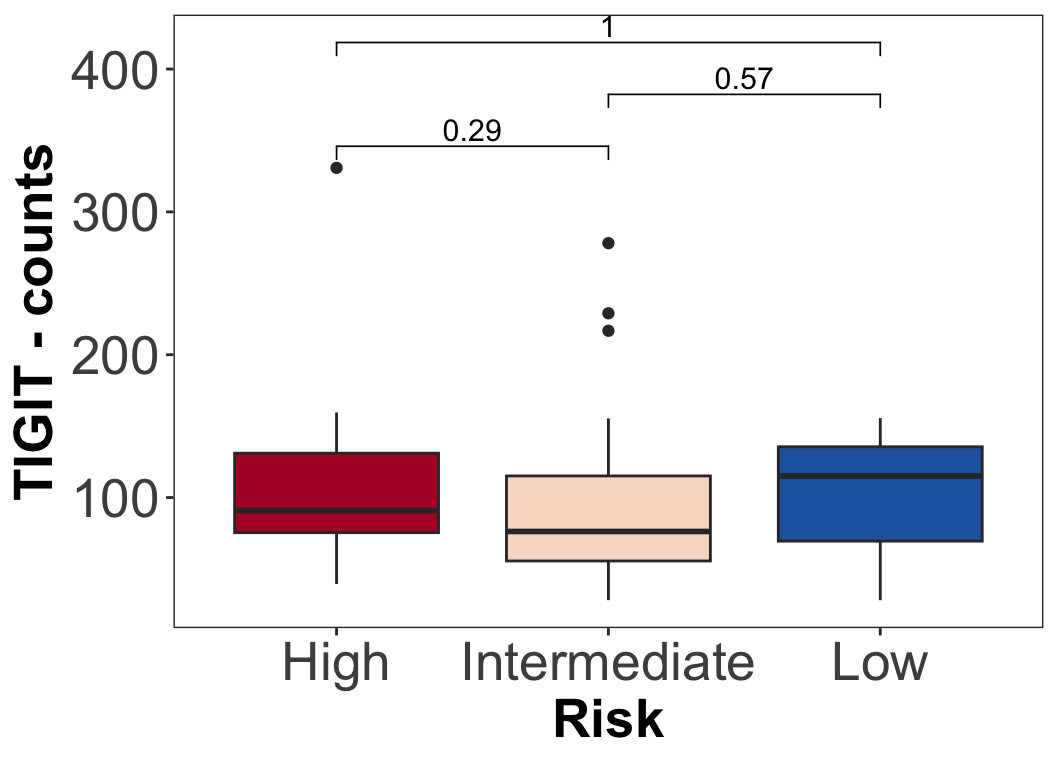

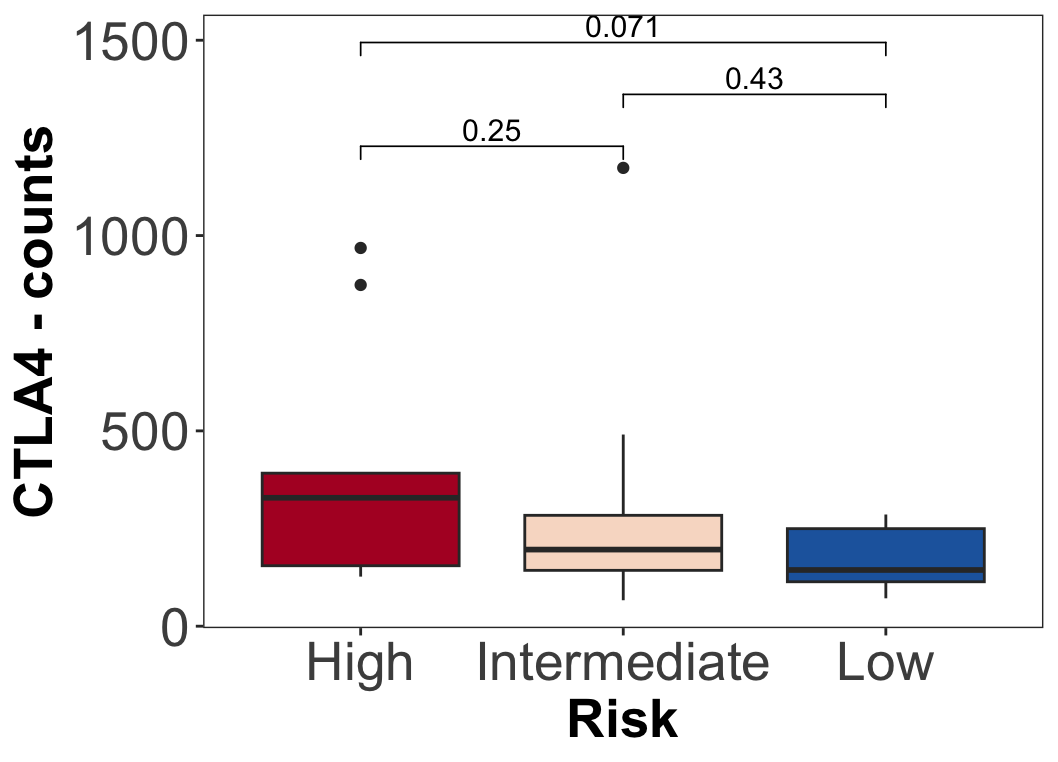

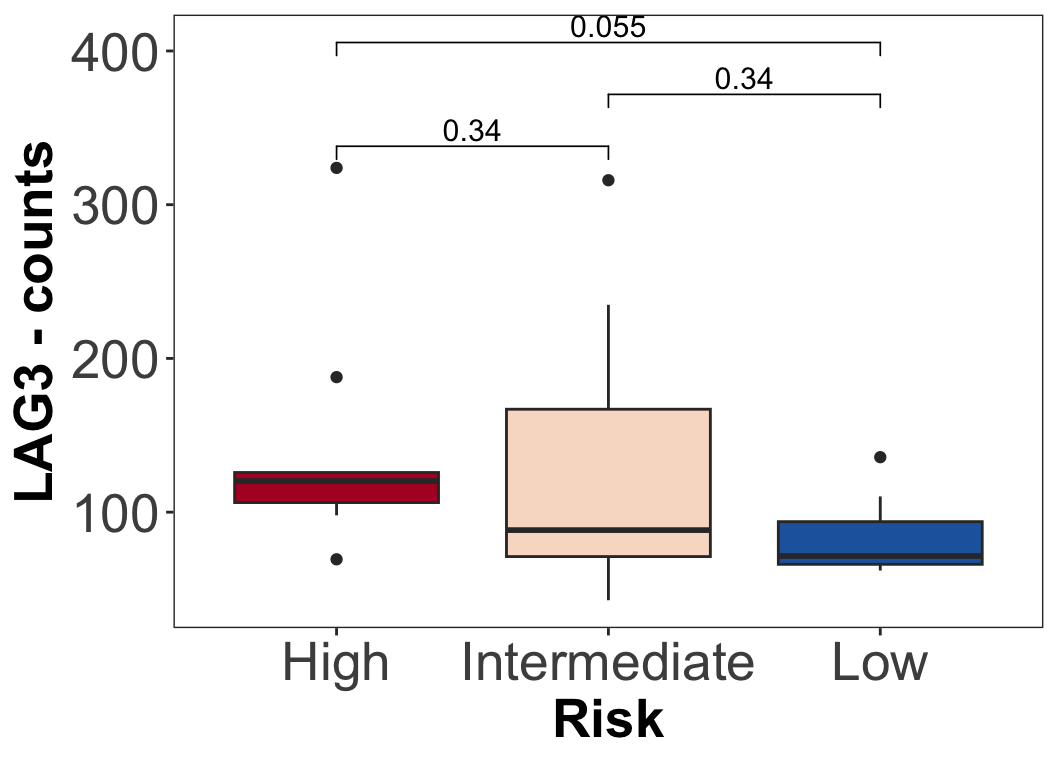

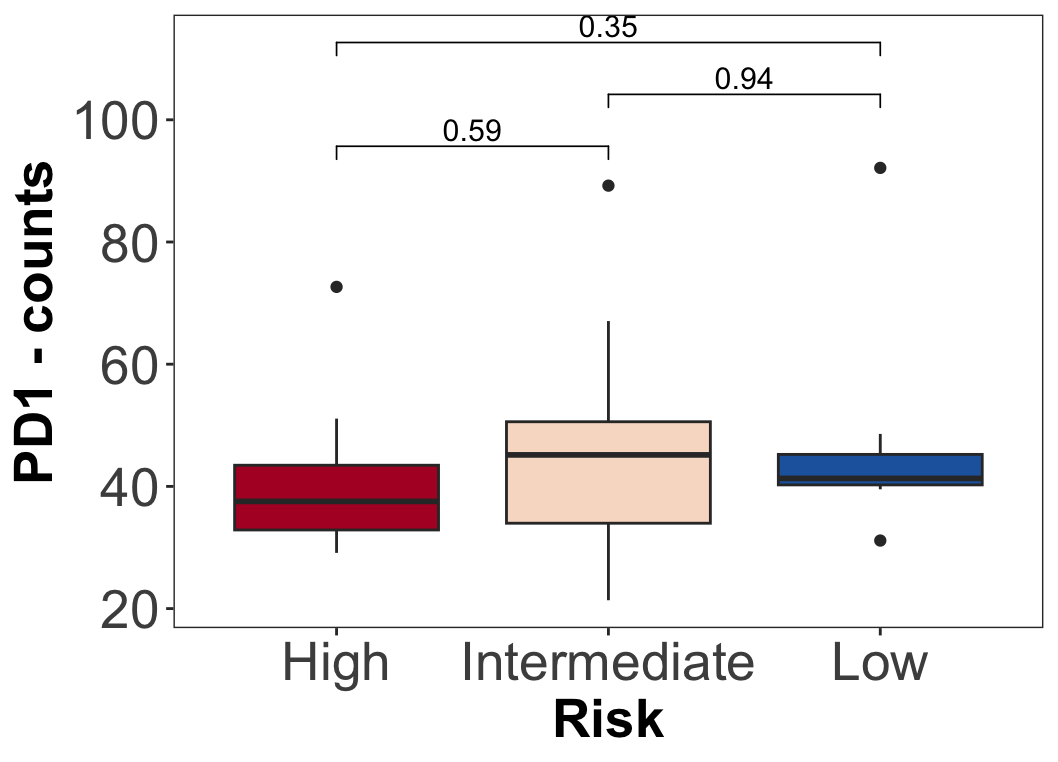

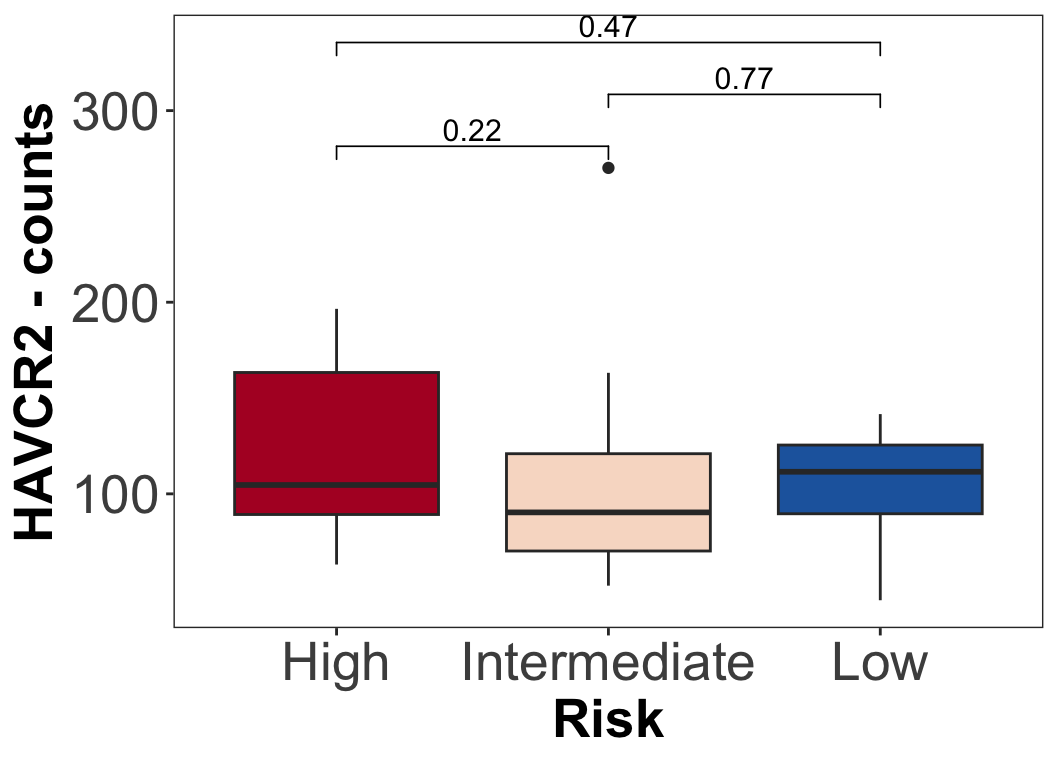


**A**

**C**

**B**

**D**

**E**

**Supplementary Figure 4.**


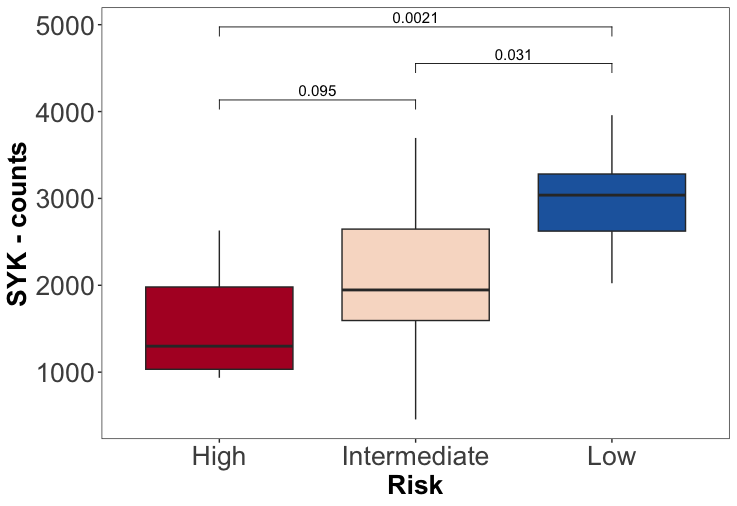

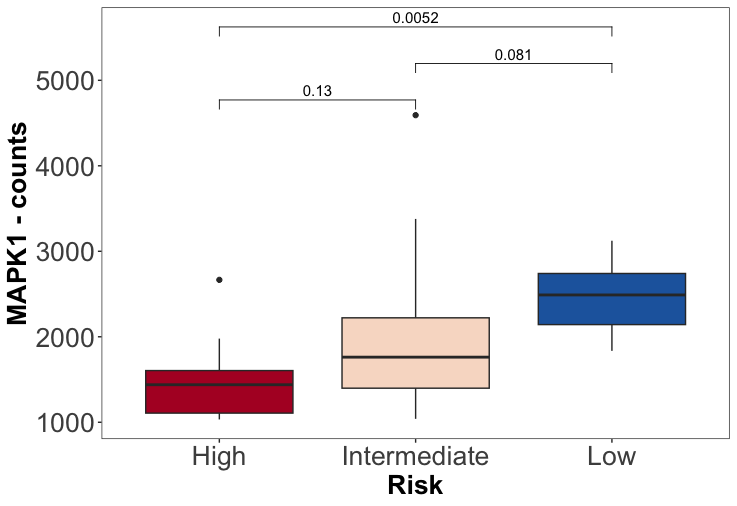


**A**

**B**
